# Cis and Trans Acting Factors Involved in Human Cytomegalovirus Experimental and Natural Latent Infection of CD14 (+) Monocytes and CD34 (+) Cells

**DOI:** 10.1371/journal.ppat.1003366

**Published:** 2013-05-23

**Authors:** Cyprian C. Rossetto, Margaret Tarrant-Elorza, Gregory S. Pari

**Affiliations:** The University of Nevada, Reno School of Medicine, Department of Microbiology & Immunology, Reno, Nevada, United States of America; Emory Vaccine Center, United States of America

## Abstract

The parameters involved in human cytomegalovirus (HCMV) latent infection in CD14 (+) and CD34 (+) cells remain poorly identified. Using next generation sequencing we deduced the transcriptome of HCMV latently infected CD14 (+) and CD34 (+) cells in experimental as well as natural latency settings. The gene expression profile from natural infection in HCMV seropositive donors closely matched experimental latency models, and included two long non-coding RNAs (lncRNAs), RNA4.9 and RNA2.7 as well as the mRNAs encoding replication factors UL84 and UL44. Chromatin immunoprecipitation assays on experimentally infected CD14 (+) monocytes followed by next generation sequencing (ChIP-Seq) were employed to demonstrate both UL84 and UL44 proteins interacted with the latent viral genome and overlapped at 5 of the 8 loci identified. RNA4.9 interacts with components of the polycomb repression complex (PRC) as well as with the MIE promoter region where the enrichment of the repressive H3K27me3 mark suggests that this lncRNA represses transcription. **F**ormaldehyde **A**ssisted **I**solation of **R**egulatory **E**lements (FAIRE), which identifies nucleosome-depleted viral DNA, was used to confirm that latent mRNAs were associated with actively transcribed, FAIRE analysis also showed that the terminal repeat (TR) region of the latent viral genome is depleted of nucleosomes suggesting that this region may contain an element mediating viral genome maintenance. ChIP assays show that the viral TR region interacts with factors associated with the pre replication complex and a plasmid subclone containing the HCMV TR element persisted in latently infected CD14 (+) monocytes, strongly suggesting that the TR region mediates viral chromosome maintenance.

## Author Summary

Human cytomegalovirus (HCMV) is a ubiquitous herpesvirus where infection is usually subclinical. HCMV initial infection is followed by the establishment of latency in CD34 (+) myeloid cells and CD14 (+) monocytes. Primary infection or reactivation from latency can be associated with significant morbidity and mortality can occur in immune compromised patients. Latency is marked by the persistence of the viral genome, lack of production of infectious virus and the expression of only a few previously recognized latency associated transcripts. Despite the significant interest in HCMV latent infection, little is known regarding the mechanism involved in establishment or maintenance of the viral chromosome. We have now identified the transacting factors present in latently infected CD14 (+) monocytes and CD34 (+) progenitor cells as well as identification of a region of the HCMV genome, the terminal repeat locus that mediates viral DNA maintenance. This is a major step toward understanding the mechanism of HCMV latent infection.

## Introduction

Human cytomegalovirus (HCMV) is a ubiquitous herpesvirus that infects 60–90% of the population and is usually subclinical, however virus infection can cause severe disease and mortality in immune compromised patients [1]. Disease manifestations include retinitis, pneumonia and hepatitis [2].

HCMV lytic phase of infection is typically studied in cell culture using human fibroblasts (HFs) and viral encoded genes are expressed in a temporally regulated manner. HCMV lytic DNA replication requires *cis* and *trans* acting factors and results in the production of infectious virus [3]–[9]. Recent high-resolution transcriptome mapping during a lytic HCMV infection, revealed a complex pattern of transcription [10]. This analysis also showed that during lytic infection most viral RNA production is concentrated in four long non-coding RNAs (lncRNAs), RNA2.7 (also known as ß2.7), RNA1.2, RNA4.9, and RNA5.0 [10]. The high expression level of viral encoded lncRNAs suggests that that these transcripts may be significant factors for regulating viral and cellular processes required for efficient viral replication.

Herpesvirus latency is defined as the persistence of the viral genome in the absence of production of infectious virus. Certain properties of latency have emerged from study of the gamma herpesviruses including maintenance of the viral chromosome is as a circular episome [11], which is controlled by virus-encoded proteins that interact with viral and host cell chromatin [12]–[23]. With respect to maintenance and replication of the HCMV DNA episome, previous studies have quantified the number of genomes during experimental and natural infection [24], [25]. However the cis acting element required for maintenance of the viral genome is unknown. Lifelong HCMV latency is established in myeloid lineage, from bone marrow-derived CD34 (+) progenitors through peripheral blood to CD14 (+) monocytes [26]–[33]. Latently infected cells contain HCMV DNA without supporting lytic replication although virus can be reactivated and recovered through differentiation [34]–[40]. The regulation and maintenance of latency is poorly understood although reactivation of latent virus is a major source of virus associated with serious disease and mortality in immunocompromised hosts, hence a better understanding of HCMV latency may lead to treatments to resolve latent HCMV genomes from infected cells. Differentiation of CD34 (+) or CD14 (+) cells, to macrophages or dendritic cells through the use of various cytokines results in reactivation and subsequent production of infectious virus [41]–[45]. Several *in vitro* experimental systems have been developed to study HCMV latency. These systems use either cultured CD14 (+) monocytes or CD34 (+) hematopoietic stem cells. CD14 (+) or CD34 (+) cells cultured *in vitro* can be infected with HCMV clinical strains, resulting in latent infection and efficient reactivation of latent virus [26], [37], [43], [46], [47].

The mechanism of HCMV latent infection is unknown and the mechanism involved in the establishment and maintenance of the latent virus genome has not been addressed in either natural or experimental models of latency. In experimental models, as well as during natural latency in CD34 (+) and CD14 (+) cells, a limited number of latency specific HCMV transcripts have been identified [41], [42], [48], [49]. Three consistently identified viral genes expressed during latency are UL138, a TNF modulator, UL111A, a variant cmvIL-10 cytokine and the UL81–82 antisense transcript encoding LUNA [41], [50], [51]. HCMV UL138 is expressed during, though dispensable for HCMV lytic replication such that UL138 mutant viruses altered latency [41], [52], [53]. This may result from the fact that UL138 has been shown to upregulate TNFR1 and sensitizes infected cells to TNFα [54], [55]. UL138 has been shown to interact with proteins encoded by nearby genes within the UL133–UL138 locus [52]. HCMV latency UL111A transcript encodes a 139 aa protein, which is a homolog to cellular IL-10 [51], [56]. Recombinant viruses that lack the ability to express UL111A are capable of establishing latency and efficiently reactivate, however latently infected cells express higher levels of surface MHC class II in the absence of UL111A expression and suggests that UL111A may function to inhibit the recognition of latently infected cells by CD4 (+) T cells [57]. The expression of LUNA during lytic infection is regulated by IE72 expression and the interaction with hDaxx [58]. LUNA has recently been implicated as having a role in virus reactivation [59].

To elucidate the factors involved in HCMV latency, we infected CD14 (+) monocytes or CD34 (+) progenitor cells with an HCMV clinical isolate [37], [46]. We have now used these experimental latency protocols, coupled with next generation sequencing, to reveal the complete high-resolution HCMV transcriptome (RNA-Seq) during early and latent infections. RNA-Seq analysis shows that during HCMV experimental and natural latency in CD14 (+) monocytes, viral transcripts encoding UL44, UL84, UL95, UL87, UL52, UL50, LUNA, UL138 and the lncRNAs 2.7 and 4.9 were detected. For latently infected CD34 (+) cells, all of the mRNAs detected in latently infected CD14 (+) cells were present, however additional transcripts encoding UL28/29, UL37/38, UL114, UL133/135 and US17 were also detected. Using chromatin isolation by RNA purification (ChIRP), lncRNA4.9 was shown to physically interact with the HCMV major immediate early promoter region and results in an enrichment of the repressive H3K27me3 mark at the MIEP during latency suggesting that HCMV latent genomes are silenced by PRC2 interaction.

To address the HCMV cis requirements for latency, we show that a plasmid containing the terminal repeat (TR) element persisted in latently infected cells, strongly suggesting that this element mediates viral genome maintenance.

## Materials and Methods

### Isolation of CD14 (+) and CD34 (+) cells from blood

Cord blood was received from the Colorado Cord Blood Bank (Univ. of Colorado). Pooled peripheral whole blood for natural infection studies was obtained from Renown Medical Center (Reno, NV) and processed with in 5 hours to isolate CD14 (+) or CD34 (+) cells. All protocols to obtain blood products were approved by IRB and Office of Human Research Protection. The pooled blood samples represented approximately 25 individual HCMV seropositive donors. Cells were isolated using human cord blood CD34 positive selection kit (Stemcell technologies) according to manufacturer's instructions. Briefly, samples were incubated with a pre-enrichment cocktail containing antibodies directed against CD66b and glycophorin A. This was step was performed for negative selection of granulocytes and erythrocytes. CD34 selected cells were retained from the non-selected cells by the use of the EasySep magnet. Cells were resuspended in culturing media or for natural infection studies immediately processed to extract total RNA. Human CD14 (+) were isolated using positive selection MACS bead and LS columns (Miltenyi Biotec) according to manufacturer's instructions and cells were resuspended in culturing media or immediately processed to extract total RNA.

Purity of isolated CD14 (+) and CD34 (+) was assessed by flow cytometry using antibodies for human CD14-FITC or CD34-FITC (Miltenyi Biotec), along with human CD45-Pacific Blue (BioLegend) for total cell staining. Isotype controls included IgG2a-FITC (Miltenyi Biotec) and IgG1-Pacific Blue (BioLegend). Approximately 0.5×10^6^ cells were stained for 30 minutes at 4°C with the fluorescently labeled antibody, washed once and resuspended in 1× PBS with 0.5%FBS, 2 mM EDTA and 1% methanol-free formaldehyde. Cells were determined to be free from red blood cells and >95% CD34 (+) or CD14 (+).

HCMV natural infection was evaluated from pooled blood from 25 seropositive individual donors. From 120 ml of pooled blood approximately 175,000 CD34 (+) cells and 32×10^6^ CD14 (+) were isolated, total RNA was extraction and DNase treated, resulting in 300 ng of RNA for CD34 (+) and 28.5 mg of RNA for CD14 (+). The RNA was used generate a sequencing library as described below.

### Culturing conditions for CD14 (+) and CD34 (+) cells

CD34 (+) cells were cultured in IMDM supplemented with 10% BIT serum substitute (StemCell Technologies), 2 mM L-glutamine, 20 ng/ml low-density lipoprotein (Sigma Aldrich), 50 mM 2-mercaptoethanol, 10 ng/ml Stem Cell factor, 10 ng/ml IL-3, 10 ng/ml G-CSF (R & D Systems) [26], [41], [60] or cultured in X-Vivo 15 (lonza) [27]. Media was refreshed every three days until latency had been established and verified. To reactive latent virus, CD34 (+) cells were stimulated to proliferate and differentiate with the addition of 10 ng/ml GM-CSF, 10 ng/ml Flt-3 ligand, 10 ng/ml TPO, and 10 ng/ml TNF for three days, followed by the addition of 50 ng/ml lipopolysaccharide (LPS) (Sigma-Aldrich) for an additional four days.

Human CD14 (+) cells purchased (Lonza and ReachBio) or isolated from cord blood were maintained in Iscove DMEM (Hyclone) supplemented with 20% heat-inactivated FBS (Atlanta Biologicals), 50 ng/mL M-CSF, 50 ng/mL stem cell factor (SCF), 50 ng/mL G-CSF, 50 ng/mL GM-CSF, 50 ng/mL IL-3 (R&D Systems) at a density of 1×10^6^ cells/mL on low cell-binding plates (Nunc Hydrocell). Medium was replaced every 3 days. BAC-derived FIX strains were propagated in human foreskin fibroblasts (HF) cells. After 12–14 days post infection cells were scraped and subjected to a freeze-thaw to release virus from the cells. Virus titer was determined with standard plaque assay on HF cells. Unless otherwise stated, infections of CD14 (+) cells were done at a multiplicity of 5 pfu/cell. Cells were incubated with virus for 1 hr. Cells were then washed twice with Hanks Balanced Salt solution (HBSS).

HCMV infected CD14 (+) or CD34 (+) cells were maintained and monitored for HCMV gene expression by qPCR analysis. Latency was determined to be established when no IE2 gene expression was detected by qPCR, detection of the virus genome and expression of LUNA and UL138. For reactivation of latent virus, CD14 (+) cells were differentiated by adherence to plastic tissue culture dishes supplemented with 100 ng/mL IL-6 (R&D Systems) at 16–18 days post infection. The reactivation from latency in either CD14 (+) or CD34 (+) was monitored for IE2 gene expression using qPCR.

For UV inactivation, 120 mls of 5×10^6^ pfu/ml of FIX BAC virus was evenly distributed in a thin layer onto a 150 mm tissue culture dish. The virus was irradiated on ice in a Stratalinker 2400 (Stratagene) for 4 min at 9.9×10^5^ mJ. UV inactivation was confirmed by the absence of immediate-early (IE) gene expression in infected human foreskin fibroblasts using qPCR.

### Co-culturing conditions of HF's and CD34 (+)

Human foreskin fibroblast cells, maintained in DMEM supplemented with 10% FBS, were plated in a 6-well tissue culture plate at 0.1×10^6^ cells per well. 24 hours after plating half of the media was removed and replaced with reactivated CD34 (+) cells (approximately 0.5×10^6^) along with its' media. Cells were co-cultured together for 10–12 days and monitored by the appearance of green plaque formation in the HF cells.

### Next generation sequencing (strand specific RNA-Seq) of experimentally or naturally infected CD14 (+) and CD34 (+) cells

Total RNA from experimentally or naturally infected (as determined above) CD14 (+) or CD34 (+) cells was isolated using PureLink RNA mini kit (Life Technology) followed by removal of genomic DNA using Turbo DNA-free (Life Technology). Poly-A RNA was enriched from total RNA by Dynabeads oligo (dT)_25_ (Life Technology) according to manufacturers instructions. The resulting Poly-A RNA was used in dUTP based NEXTflex Directional RNA-Seq Kit with Illumina compatible adaptors (Bioo Scientific) according to manufacturers instructions. The resulting libraries were verified on a Bioanalyzer High sensitivity DNA chip (Agilent) and quantified with real-time PCR using Illumina compatible kit and standards (KAPA). Final libraries were sequenced using an Illumina MiSeq instrument. HCMV transcript discovery and alignment was performed using CLC Genomics Workbench software and strand specific RNA-Seq parameters. Transcripts were aligned to the Fix strain (VR1814) reference genome.

### Chromatin Isolation by RNA purification (ChIRP)

Tiling of RNA 4.9 with biotin labeled DNA probes retrieves specific RNA 4.9 bound proteins and DNA sequences. Original protocol is from Chu et al [61]. All probes were biotinylated at the 3′ end with an 18-carbon spacer arm; probes were designed against RNA 4.9 full-length sequence using an online designer at http://www.singlemoleculefish.com, and synthesized at Protein and Nucleic Acid Facility (Standford University). Samples were processed as described previously [62]. Eluted DNA was resuspended in 50 µl of water and used for end point PCR. Primers for the PCR include MIEP-1 forward: GTGTTTGTCCGAAATACGCG, reverse: GCCTCATATCGTCTGTCACC; MIEP-2 forward: GTTACATAACTTACGGTAAATGGCC, reverse: CCAAAACCGCATCACCATG; MIEP-3 forward: GATTTCCAAGTCTCCACCCC, reverse: GCGGTACTTACGTCACTCTTG; MIEP-4 forward: CCCCGCTTCCTTATGCTATAG, reverse: AAGAACCCATGTCCGGAAC; MIEP-5 forward: CTCCTTGCTCCTAACAGTGG, reverse: GTACTGCTCAGACTACACTGC; UL19 forward: CCTGTATGAGCTGTTTCGACG, reverse: GACTCACATCTAGCTCGTCTTC. ChIRP PCR primers are shown in Table S2 in Text S1.

### Supernatant virus detection by real-time PCR

For CD14 (+) and CD34 (+) culturing media was changed every three days in order to replenish nutrients and cytokines necessary to maintain the cell in culture. The supernatant that was removed from the cells was collected and used for quantitative real-time PCR to obtain relative values for the amount of virus being produced. The supernatant was subjected to two low speed spins, 400×g for 10 minutes in a table top centrifuge to remove any residual cells. For the real-time PCR reaction 5 µl of supernatant was used in a total reaction volume of 20 µl using Taqman Universal 2× master mix (Life Technology) and 20× primer-probe (IDT). Relative Ct values of viral supernatant DNA were correlated to standard curve from a known concentration of purified FIX-BAC DNA.

### Trypan blue exclusion test of cell viability

Cell viability, density and growth was monitored by using trypan blue exclusion test. Briefly, cells were gently resuspended and a small aliquot was removed. A 1∶1 dilution of cell suspension and 0.4% Trypan Blue solution (MP Biomedicals) was counted in a hemacytometer to determine and monitor total number of live cells.

### H3K27me3/H3K4me3 ChIP and quantitative real-time PCR analysis

Evaluation of changes in histone modifications was performed as previously described [62]. Fold-enrichment of histone marks at various genomic loci was calculated as IgG-subtracted %Input of the locus normalized by the IgG-subtracted %Input of the reference gene GAPDH. 3 separate experiments were performed. PCR primers are listed in Table S4 in Text S4.

### Transfection of demethylases JMJD3 and UTX into latently infected CD14 (+) cells

5×10^6^ latently infected CD14 (+) cells were transfected with 2.5 µg each of JMJD3-HA and UTX-HA expression plasmids (Addgene), or 5 milligrams of GFP-control plasmid, and a non-transfected control group. The cells were transfected using Nucleofector device and Amaxa Human Monocyte Nucleofector kit (Lonza) according to manufactures instructions. Transfection efficiency was monitored by GFP expression. 72 hours post transfection RNA and protein was harvested using RNA/DNA/Protein purification kit (Norgen BioTek Corp.). Total RNA was subjected to removal of genomic DNA using Turbo DNA-free (Life Technology) according to manufacturers instructions. The purified RNA was then used for cDNA synthesis as previously described. The resulting cDNA was quantified using real-time PCR and Taqman primers targeting specific HCMV transcripts.

Expression of UTX and JMJD3 was confirmed by Western blot where total cell protein extracts were resolved by SDS-PAGE gel that was subsequently transferred to a polyvinylidene difluoride (PVDF) membrane, blocked with 5% nonfat dry milk powder in 1× TBST buffer and reacted with antibodies specific for HA-tag (Sigma-Aldrich). After one hour incubation with the primary antibody the membrane was washed in 1× TBST three times 5 minutes each. The secondary antibody donkey anti-mouse IgG conjugated to Alexa Fluor-680 (Life Technology) was diluted in blocking buffer and added to the membrane for 30 minutes as which time it was again washed with 1× TBST three times 5 minutes each. Specific proteins bands were visualized using the Odyssey by LI-COR.

### Real-time PCR (qPCR)

Total RNA was isolated from cells using PureLink RNA mini kit (Life Technologies), followed by removal of genomic DNA using Turbo DNA-free (Life Technologies). cDNA was synthesized from 1 µg of total RNA in the presence of random hexamers, dNTPs, and Superscript III reverse transcriptase (Life Technologies). The resulting cDNA was then used along with Taqman Universal PCR Master Mix (Life Technologies) and specific primers and FAM labeled probes (IDT) in an Eppendorf RealPlex. The following real-time PCR program was used: one cycle 95°C hot start for 5 minutes, and forty cycles of 95°C for 15 seconds and 60°C for 1 minute. Primers used for detection of specific gene expression are shown in Table S3 in Text S1.

### UL84 and UL44 ChIP-Seq

4–5×10^6^ latently infected CD14 (+) monocytes were cross-linked by the addition of formaldehyde to a final concentration of 1%, incubated at room temperature for 10 minutes and quenched with 0.125 M glycine. After washing with ice cold PBS, cells were lysed in 1 ml/5×10^6^ cells ice-cold lysis buffer (0.5% NP-40, 150 mM NaCl, 50 mM Tris, pH 7.4, 1 mM EDTA, and protease inhibitors). Lysed cells were dounced followed by collection of the crude nuclear extract by centrifugation. The nuclear pellet was resuspended in 1 ml of RIPA (50 mM Tris, pH 7.4, 150 mM NaCl, 1% Triton-X, 0.1% SDS, 0.5% sodium deoxycholate, 1 mM EDTA) and sonicated with a Fisher Scientific Sonic Dismembrator and micro-tip at 40% amplitude for 40 cycles of 20 s on followed by 20 s off in a wet ice bath. The sonicated chromatin was collected by centrifugation at 20,000×g for 15 min at 4°C to remove cellular debris. Chromatin shearing to 150–200 bp fragments was confirmed by agarose gel electrophoresis. 100 ul of chromatin was reserved for input library preparation and the remainder was pre-cleared for 1 hour at 4°C with 100 ul normal mouse IgG sepharose beads followed by incubation overnight with 20 ug UL84 or 100 ug UL44 mAb. 200 ul of Active Motif magnetic IgG coated beads were prepared by blocking overnight with 5 mg/ml BSA and 200 ug/ml yeast tRNA (Ambion) in PBS. Beads were washed once with 5 mg/ml BSA in PBS and added to immunoprecipitated chromatin. Immunoprecipitation was performed for 4–5 hours at 4°C. The beads and immunoprecipitated complexes were washed at room temperature with rotation twice for 1 min with RIPA, five times for 5 minutes each with LiCl_2_ buffer (500 mM LiCl2, 100 mM Tris pH 7.4, 1% NP-40, 1% sodium deoxycholate), and one brief wash with TE (10 mM Tris, pH 8.1, 1 mM EDTA). DNA was eluted from the beads by the addition of 200 ul elution buffer (1% SDS, 0.1 M NaHCO_3_) and reversed cross-linked overnight at 65°C. Reserved input DNA was also reverse cross-linked and treated the same as ChIP samples from this point forward. Reversed cross-linked UL84 ChIP DNA or UL44 Chip DNA and respective Input DNA were purified with a Qiagen min-Elute kit. Purified DNA was quantified using an Invitrogen Qubit Flourometer and sequencing libraries were created from 10 ng of ChIP or Input DNA with a Bioo Scientific NEXTflex ChIP-Seq library preparation kit (#5143-01). Library integrity was analyzed with an Agilent 2100 Bioanalyzer and quantified using a KAPA q-PCR kit. 12 pM each of UL84 ChIP or UL44 ChIP library and 6 pM of respective Input library were sequenced on an Illumina MiSeq. ChIP-Seq data analysis, including read mapping to FIX genome (VR1814) and peak calling was performed using CLC Genomics Workbench with a maximum false discovery (FDR) rate of 5%, a window size of 250 bp, and reads were shifted based on a length of 200 bp. The data is the result of four separate experiments.

### Reverse Transcription PCR (RT-PCR)

10×10^6^ CD14 (+) or CD34 (+) cells were infected with FIX virus and cells were monitored for IE gene expression using qPCR. Once latency had been established, approximately 18 dpi, total RNA was isolated from cells using PureLink RNA mini kit (Life Technologies), followed by removal of genomic DNA using Turbo DNA-free (Life Technologies). The resulting RNA was used with Qiagen OneStep RT-PCR kit with primers designed to mRNA shown in Table S3 in Text S3.

### RNA Cross-linking Immunoprecipitation (RNA CLIP)

20×10^6^ CD14 (+) cells were infected with FIX virus. 18 days post infection cells were harvested and processed as described previously [62]. RNA-protein complexes were precipitated by adding 300 µl lysate, 5 µl antibody, UL84, UL44, SUZ12 (Active motif, cat. # 39357), EZH2 (Active motif, cat. # 39875) or GAPDH (Abcam, ab128915), 25 µl Protein G magnetic beads (Active Motif), and 1 µl RNase Out. Primers used for PCR are shown in Table S1 in Text S1.

### Transfection of CD14 (+) monocytes and Gardella gels

CD14 (+) monocytes were infected with BAC-derived FIX virus at a multiplicity of 5 pfu/cell. Cells were maintained and monitored for HCMV gene products by qPCR analysis. Latency was determined to be established when no IE gene expression was detected by qPCR analysis. After established latency pGEM7zf(−), pGEM-oriLyt, or pGEM-TR plasmids (4 ug) were added to 3×10^6^ CD14 (+) latent or control non-infected cells using Nucleofector 2b device and Human Monocyte Nucleofector kit (Lonza) according to manufacture's instructions for program Y-001 on the device. Transfected cells were immediately transferred to 12-well low cell-binding plates (Nunc Hydrocell) containing 37°C prewarmed media and placed back into 37°C/5% CO_2_ incubator. Cells were evaluated for viability using trypan blue and for transfection efficiency using a spike of a plasmid expressing EGFP. Cells were determined to be 80% viable and a transfection efficiency of over 70% was achieved. For analysis of transfection input, 24 hrs post transfection total DNA from 2×10^5^ cells was harvested using Norgen DNA/RNA/Protein purification kit according to manufacture's instructions. DNA was eluted from the column in 100 mls of DNA elution buffer, 50 mls was then used for EcoRI (New England Biolabs) restriction digest. The total sample of EcoRI digested DNA was loaded onto a 0.8% 1× TAE (40 mM Tris-HCl, 20 mM Acetic Acid, 1 mM EDTA) agarose gel and electrophoresed in 1× TAE buffer until the dye front reached the bottom of the gel. For analysis of plasmid maintenance, approximately 2 million latently infected and transfected (15 dpt) CD14 (+) cells/well were prepared, loaded, and electrophoresed on Gardella gels as previously described [63]. DNA was transferred to Zeta-Probe membrane (Bio-Rad) by the alkaline transfer method according to the manufacturer's instructions and hybridized to using a ^32^P-labled pGEM probe. Membranes were hybridized with 5 ng of probe in 10 ml of hybridization buffer (1.5× SSPE, 1 mM EDTA, 7% sodium dodecyl sulfate [SDS], 10% [wt/vol] polyethylene glycol) for 16 hrs at 65°C in a hybridization oven (Robbins Scientific). Post-hybridization washed were performed with 2× SSC and 0.1% SDS twice for 15 minutes each at 65°C, and then with 0.1× SSC and 0.1% SDS twice for 30 minutes at 65°C. Southern blot were exposed and visualized using the Storm Scanner imaging system (GE Healthcare).

### FAIRE-Seq (Formaldehyde Assisted Isolation of Regulatory Elements)

FAIRE was performed as previously described [64]. Briefly, 3×10^6^ CD14 (+) monocytes infected with HCMV strain FIX at 4 dpi or 18 dpi were cross-linked with formaldehyde and lysed, followed by sonication to shear chromatin. 10% of the sheared cross-linked chromatin was reserved for input and the remainder was extracted twice with phenol∶chloroform to remove regions of DNA associated with nucleosomes. The aqueous layer containing open regions of the chromatin corresponding to nucleosome depletion was reserved, reverse cross-linked, and further purified by ethanol precipitation. Input DNA was also reverse cross-linked and purified by ethanol precipitation. Sequencing library preparation was performed using an Illumina Tru-Seq DNA library kit. Input and FAIRE libraries were sequenced by the University of California-Irvine High Throughput Genomics Facility on an Illumina Hi-Seq 2000. Approximately 60 million reads per library were generated. Read alignment with FIX strain (VR1814) was done using CLC Genomics Workbench.

### Cloning of HCMV terminal repeat

PCR primers were designed to specifically amplify the terminal repeat region using FIX BAC DNA as a template and PrimeSTAR GXL DNA polymerase (Takara). After PCR was completed the product was precipitated with 1/10 volume 3 M NaAcetate and 2.5 X volume 100% ethanol. The DNA pellet was resuspended in 100 mls of water and 100 mls of 2× Easy-A Master mix (Stratagene) was added. The DNA was incubated at 70°C for 30 minutes and then precipitated with 1/10 volume 3 M NaAcetate and 2.5 X volume 100% ethanol. The DNA pellet was resuspended in 20 µl of water and 3 µl was used in pGEM T-easy (Life Technology) cloning reaction according to manufacturers instructions. The resulting clones were subjected to restriction enzyme digestion and DNA sequencing to determine clones with the correct insert.

### Chromatin immunoprecipitation assay (ChIP) to identify licensing factor interaction with HCMV TR/IR regions

Each ChIP was performed with chromatin from approximately 200,000 latently infected CD14 (+) monocytes. DNA-protein complexes were immunoprecipitated using CDT1 (Abcam, ab70829), MCM3 (Abcam, ab4460), or an isotype control antibody. PCR for TR region was performed using PrimeSTAR GXL DNA Polymerase (Takara). Primers to detect the TR region were: Set #1 Forward 5′-ACA CCT CCG ACG TCC ACT ATA TAC CA-3′/Reverse 5′-CGT CCA CAC ACG CAA CTC CAA TTT-3′ or Set #2 Forward 5′- GGT GAC GTC GGA GAC AGG G-3′/Reverse 5′- TGC TGT GGT GTA AGG GTA AGG TGT-3′.

### PCR amplification of genomic DNA from latently infected CD14 (+) monocytes

Genomic DNA was isolated from approximately 2 million mock infected, 18 dpi, and 20 dpi FIX BAC latent CD14 (+) monocytes using a Norgen DNA/RNA/Protein purification kit. Primers amplifying a region within the TR or IR were Forward: 5′-aacgacagacgaagtacggcacaa-3′ and Reverse 5′-acaaacaccgcagaactccttgacg-3′. Takara PrimeSTAR GXL polymerase was used for PCR.

## Results

### High-resolution transcriptome mapping for latent HCMV infection in CD14 (+) monocytes and CD34 (+) hematopoietic stem cells (RNA-Seq)

Although previous studies investigated viral gene expression in HCMV latently infected CD14 (+) and primary hematopoietic progenitor cells [26], [41], [42], [46], [48], [49], [65], [66], new technologies now allow for the high-resolution evaluation of the HCMV latent virus transcriptome. While there is little or no understanding with respect to the mechanism involved to maintain or replicate the virus genome in a latent environment, we speculate that viral encoded factor(s) play a significant role in viral DNA maintenance. This assumption is solidly based on other herpesvirus systems, the most prominent being the gamma herpesviruses where at least one virus encoded factor is required for viral genome maintenance and replication [14], [15], [67]–[71]. Hence, the first step in identifying likely candidates involved in viral genome maintenance/replication is to identify all viral encoded transcripts present during latent infection.

**Figure 1:**
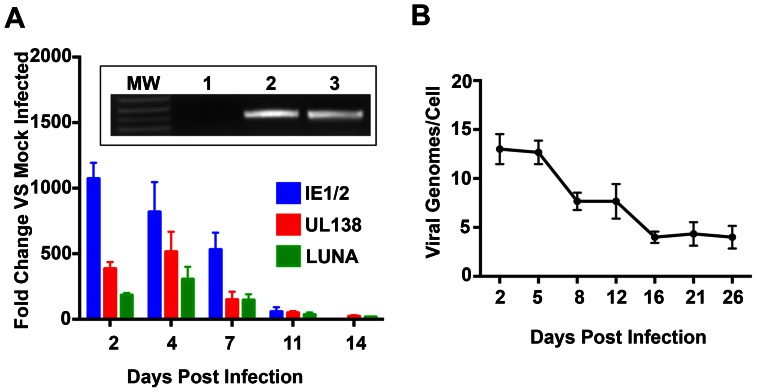
Infected CD14 (+) cells express latency associated transcripts and stable DNA copy number after 14 days in culture. (A) RT-qPCR analysis for the expression of IE1/IE2, LUNA and UL138 mRNA in infected CD14 (+) monocytes. CD14 (+) cells were infected with FIX BAC virus and total cellular RNA was harvested various days post infection and subjected to RT-qPCR using TaqMan primers and probes specific for IE1 and IE2 mRNA. Inset figure: Detection of viral genomic DNA in infected CD14 (+) monocytes by PCR. Lanes: 1, uninfected; 2, 5-day post infection; 3, 18 day post infection. Primers used were specific for the TR region of the genome. (B) CD14 (+) monocytes infected cell viral genome copy number. Cells were harvested at various days post infection and subjected to qPCR using primers and probes specific for the HCMV viral chromosome. Absolute viral genome copy number was determined by comparing to a standard curve and calculated on a per cell basis. Error bars are the standard deviation of the mean for 4 separate wells.

CD14 (+) monocytes were cultured as previously described using specific growth media and culture plates that did not allow attachment/differentiation of cells [46]. Undifferentiated CD14 (+) monocytes were infected with the FIX BAC clinical isolate virus strain and viral immediate early (IE1/IE2), LUNA and UL138 transcript levels were measured at various days post infection using qPCR (Fig. 1). Immediate early mRNA was detected at days 2, 4, 7 and 11 days post infection (Fig. 1). Immediate early gene expression was no longer detected by 14 days post infection (Fig. 1) suggesting that HCMV virus genomes were not producing immediate early proteins and lytic replication had concluded. LUNA and UL138 transcripts were still detected, indicating that the virus entered the latent phase (Fig. 1). HCMV genomic DNA was measured from infected cells at 5 and 18 days post infection by qPCR. The HCMV genome was present in latently infected cells at approximately 4 copies per cell at 18 days post infection. Viral DNA was also detected using traditional PCR at 5 and 18 days post infection (Fig. 1, inset). We also measured viral genome copy number over a 26-day period of infection (Fig. 1B). Viral genomes were present at approximately 4 copies per cell during latent infection at 16 days post infection (Fig. 1B). Cells were monitored during infection and analyzed to ensure the absence of macrophage or dendritic cell markers (CD11c+, CD11b+, CD141+, CD303+, CD11b+, CD68+, CD80+).

**Figure 2:**
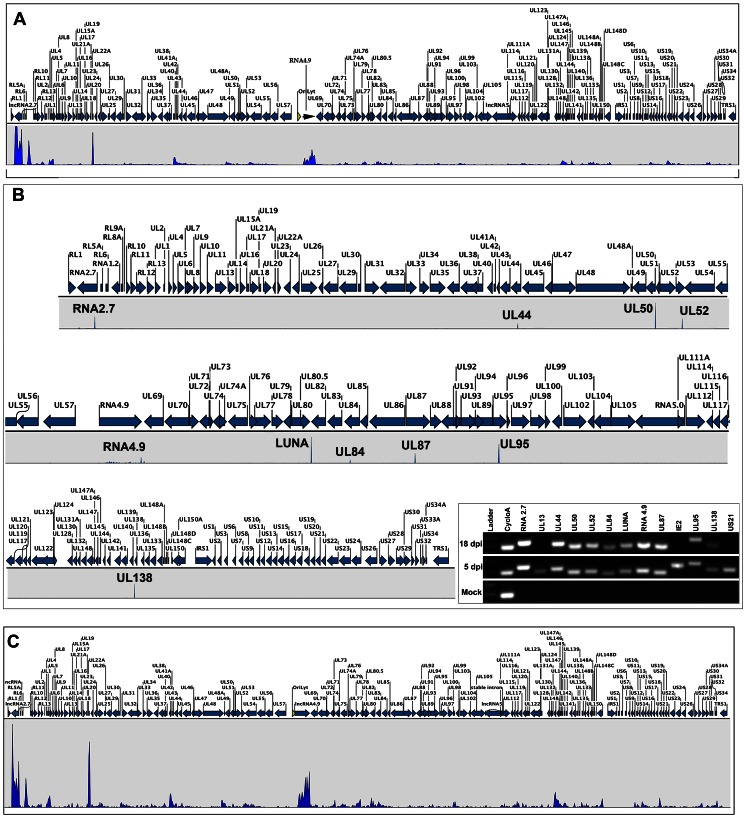
RNA-Seq analysis of HCMV infected CD14+ cells. (**A**) HCMV transcriptome 5 days post infection. (**B**) HCMV transcriptome 18 days post infection. Transcript analysis was performed using CLC Bio Genomics Workbench software. Peaks were calculated and determined based on 4 independent experiments and using a minimum of 20 read cut off and pValue of <0.05 was used to determine peaks shown. Peaks identified from 18 day CD14 (+) latent infection were: UL138, UL95, UL87, UL84, UL52, UL50, UL44, LUNA, RNA4.9 and RNA2.7. Peak heights are indicative of relative transcript accumulation in infected cells. Arrows indicate direction of transcripts. **Inset**: RT-PCR evaluation of identified ORFs during lytic or latent infection. Negative controls for latent infection: IE2, UL13 and US21. CycloA = cyclophillin A. (**C**) HCMV transcriptome upon reactivation of latently infected CD14 (+) monocytes with treatment of IL-6 and culturing cells on a surface that allows for cell attachment.

For RNA-Seq, total cellular RNA from latently infected CD14 (+) monocytes was extracted at 5 and 18 days post infection. Total cellular RNA was also extracted from HCMV latently infected CD14 (+) monocytes, after an 18-day incubation subsequent to treatment with IL-6 and re-plating of cells on a surface that allowed attachment. This lytic virus reactivated sample, along with the 5 and 18 day samples were subjected to next generation sequencing (RNA-Seq). RNA-Seq was performed using an Illumina HiSeq 2000 instrument (54 million reads per sample, paired end directional sequencing). Data was analyzed using CLC Bio Genomics Workbench software (RNA-Seq analysis) using mock-infected RNA as a control, 4 independent biological replicates were used for analysis. Figure 2 is a “peak map” showing the location and relative abundance of transcripts from the HCMV FIX BAC viral genome identified by RNA-Seq. The height of the peaks represents the relative number of reads for the transcripts detected and correlates to the relative abundance of the mRNA in cells. At 5 days post infection almost all (99%) of the HCMV ORFs present within the viral genome were expressed (Fig. 2A). Interestingly, and partly consistent with what was previously reported from an RNA-Seq analysis of HCMV lytic infection, the highest amount of transcript accumulation after a 5-day infection of CD14 (+) monocytes occurred from the expression of UL22A, lncRNAs 2.7 and 4.9 loci (Fig. 2A). UL22A is expressed in HCMV infected dendritic cells during lytic infection but has not been previously described in early infection of CD14 (+) monocytes. UL22A is responsible for immune evasion and selectively blocks CCL5 [72]. Immediate early transcripts encoding IE1/2 (UL122–123) were also detected as well as all early transcripts encoding replication proteins (UL44, UL70, UL105, UL102, UL54 and UL57) (Fig. 2A). Late transcripts encoding viral glycoproteins and capsid proteins were also present at 5 days post infection.

At 18 days post infection of CD14 (+) monocytes the transcriptome analysis showed that only a subset of viral-encoded RNAs were present in latently infected cells and confirmed that neither immediate early IE2 or IE1 mRNAs were expressed, nor were any other immediate early transcripts detected (Fig. 2B). Transcripts were detected encoding LUNA, UL95, UL138, UL87, UL84, UL52, UL50 and UL44 along with transcripts for the long noncoding RNAs RNA2.7 and RNA4.9 (Fig. 2B). Very low levels of UL111A mRNA were detected, which cannot be seen on the peak map shown (cutoff was 20 reads). A list of all transcripts detected during latent infection is shown in table 1 along with the number of reads from the RNA-Seq analysis. These data are the first reporting of the total virus encoded transcriptome from latently infected CD14 (+) monocytes using next generation sequencing. To confirm the presence of RNA transcripts we performed reverse transcriptase PCR (RT-PCR) using RNA isolated from 5 and 18 day infected CD14 (+) monocytes and primers specific for each of the transcripts detected as well as control transcripts. All transcripts identified from RNA-Seq analysis were detected using RT-PCR, whereas we were unable to detect IE2, UL13 or US21 mRNAs (Fig. 2B, Inset figure), confirming the results from the next generation sequencing.

**Table 1:** CD14 HCMV latent experimental infection.

Gene	Reads
LUNA	450
UL95	356
UL50	321
RNA4.9	309
RNA2.7	254
UL52	187
UL138	126
UL87	107
UL84	79
UL44	30
UL79	16
UL111A	8

Lytic reactivation of latent virus was also performed by incubating latently infected (18 days post infection) CD14 (+) monocytes with IL-6 and re-plating in tissue culture flasks that allowed for cell attachment. After a 7-day incubation with IL-6, total cellular RNA was harvested and subjected to next generation sequencing. RNA-Seq analysis showed that HCMV gene expression was observed from almost the entire viral genome consistent with lytic virus replication (Fig. 2C). Together these data show that HCMV latent infection in CD14 (+) monocytes is characterized by the absence of transcripts encoding immediate early proteins and the expression of a several specific transcripts, some of which were normally associated with lytic DNA replication and expression of two lncRNAs.

**Figure 3:**
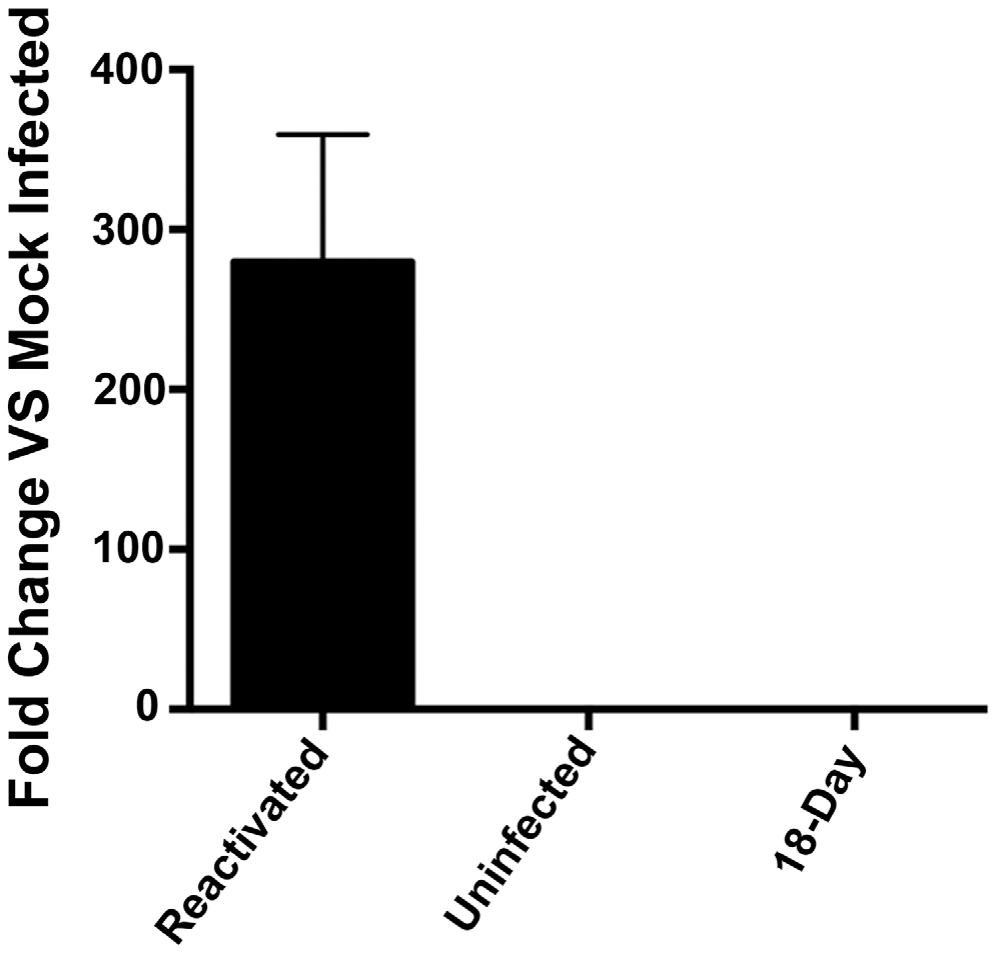
Detection of supernatant virus from reactivated CD14 (+) monocytes. Supernatants were collected from CD14 (+) monocytes infected with HCMV after 18 days post infection as well as from cell reactivated after 18 days in culture. Uninfected supernatants were also collected. qPCR was performed using primers and probes specific for the UL54 and UL95 loci. A total of 6 wells (3 for each probe) for each sample were collected and error bars are the standard deviation from the mean from 6 replicates.

To test for the production of virus, supernatants were collected at 18 days post infection from reactivated samples and measured the amount of HCMV DNA by qPCR. In reactivated cells, supernatant virus was detected indicating that lytic virus infection was efficiently reactivated in these cells (Fig. 3).

**Figure 4:**
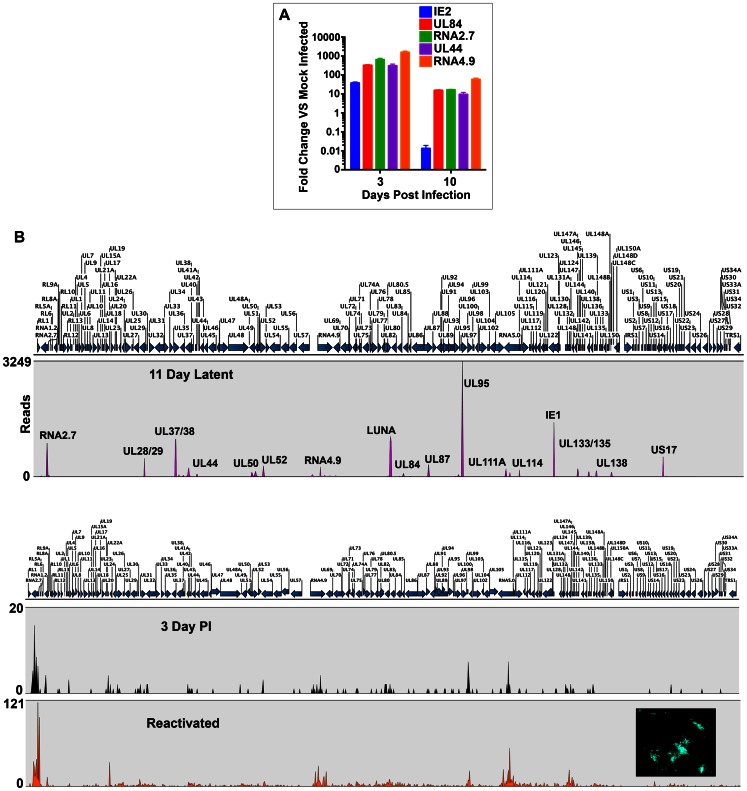
RNA-Seq analysis of HCMV infected CD34 (+) cells. Latent transcriptome from CD34 (+) cells shares core transcripts with those observed from CD14 (+) latent infection. (A) qPCR evaluation of specific gene expression from CD34 (+) cells infected with FIX BAC virus at 3 and 10 days post infection. (B) RNA-Seq detection of transcripts from infected CD34 (+) cells during latent (11 days post infection), 3 days post infection and from cells reactivated after 11 days post infection. Peaks were calculated and determined based on 3 independent experiments and using a minimum of 20 read cut off and pValue of <0.05 was used to determine peaks shown. Peak maps are shown where the number of transcript reads is shown to the left of the graphs. CD34 (+) cells latency-associated transcripts identified were: RNA2.7, UL28/29, UL37/38, UL44, UL50, UL52, RNA4.9, LUNA, UL84, UL87, UL95, UL111A, UL114, IE1, UL133, UL135, UL138, US17.

For CD34 (+) cells, we evaluated mRNA expression at 3 days post infection as well as at a time point when IE2 transcripts were no longer detected. RNA was harvested at 3 and 10 days post infection and qPCR was performed. We measured the transcript abundance of IE2, UL44, UL84, RNA 2.7 and RNA4.9. At 10 days post infection of CD34 (+) cells, no IE2 mRNA was detected, however transcripts for UL44, UL84 and the two lncRNAs were detected (Fig. 4A). Based on the results of the qPCR showing that latency associated genes were expressed in the absence of detectable IE2, we harvested total cellular RNA and performed next generation sequencing. The results of the RNA-Seq are shown in figure 4B. For HCMV latent infection of CD34 (+) cells we harvested RNA at 11 days post infection and the RNA-Seq peak map shows the presence of the same transcripts detected in latently infected CD14 (+) cells (Fig. 4A, 11 day latent). Interestingly, several additional transcripts were detected. RNA-Seq of latently infected CD34 (+) cells shows that the mRNAs for UL28/29, UL37/38, UL114, IE1 UL133/135, UL111A and US17 were detected (Fig. 4A). Hence these data show that there are common transcripts associated with HCMV latent infection in both CD14 (+) and CD34 (+) cells, including two lncRNAs and mRNAs that encode UL84 and UL44. At 3 days post infection less than half of the total HCMV genes were expressed and at a very low levels (Fig. 4B, 3 day PI). In reactivated cells, the entire HCMV genome was activated and the relative amount of transcription was about 5-fold higher (Fig. 4B, reactivated). Cells were co-cultured with HFFs and cells were observed for the formation of green plaques. Green plaques were readily visible after a 12-day incubation indicating that efficient reactivation from latent infection was achieved (Fig. 4B, reactivated inset image of green plaques on HFF cells).

We also evaluated transcripts present in naturally latent infected CD14 (+) monocytes and CD34 (+) cells isolated from whole blood from pooled HCMV positive donors (Renown Hospital, Reno NV). Cells were isolated, RNA was extracted and next generation sequencing libraries were generated. RNA-Seq analysis performed on naturally latent infected cells showed that, although the relative abundance of individual transcripts varied between experimental and natural infection, the transcripts present were mostly consistent with experimental latency models (tables 1 and 2, tables 3 and 4). These data strongly suggest that the most of the transcripts detected during experimental latency in both CD14 (+) and CD34 (+) cells mirrors an HCMV natural latent infection and consequently validates *in vitro* HCMV latency infection protocols used for this study.

**Table 2:** CD14 HCMV natural latent infection.

Gene	Reads
RNA2.7	504
LUNA	453
UL95	343
UL44	245
UL50	187
UL87	123
RNA4.9	104
UL52	54
UL79	44
UL84	36
UL138	24

**Table 3:** CD34 HCMV latent experimental infection.

Gene	Reads
UL95	3249
IE1	1532
UL37/38	1125
LUNA	1098
RNA2.7	965
UL28/29	643
US17	602
RNA4.9	321
UL87	159
UL52	105
UL50	67
UL111A	55
UL114	43
UL133/135	40
UL138	39
UL84	20
UL44	20
UL79	18

**Table 4:** CD34 HCMV natural latent infection.

Gene	Reads
UL87	15,297
UL95	2,154
UL138	1,645
UL79	1,389
LUNA	1,347
UL126a	976
UL50	758
UL84	730
UL52	662
UL44	213
RNA4.9	75
RNA2.7	39
IE1	21
UL28/29	11
UL133	5

### HCMV UV inactivation abrogates latent virus gene expression

**Figure 5:**
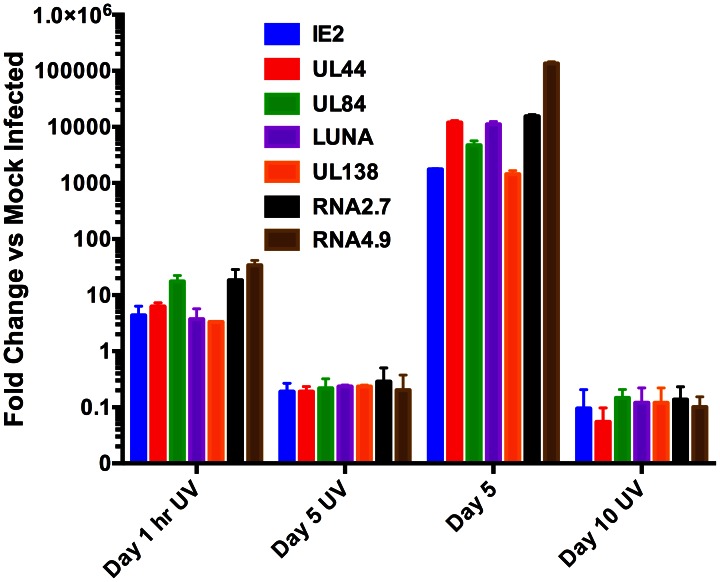
UV inactivation of virus abrogates accumulation of IE2 and latency associated transcripts in CD14 (+) monocytes. CD14 (+) monocytes were infected with either wt FIX BAC virus or UV inactivated virus. Total cellular RNA was harvested at 1 hr, 5 and 10 days post infection and qPCR was performed to detect transcripts IE2, UL44, UL84, LUNA, UL138, lncRNA 2.7 and lncRNA4.9. Transcript fold increase was determined using mock infected as reference. 3 separate experiments were preformed and error bars are the SD of the mean.

Since we observed robust transcription at 5 days post infection of CD14 (+) monocytes, we wanted to determine if virion associated transcripts could be detected from UV inactivated virus at 5 days post infection and evaluate the ability of virus encoded mRNAs to persist in CD14 (+) monocytes. It was previously described that HCMV virions contain virus-encoded mRNAs [73], [74]. Hence we infected CD14 (+) monocytes with UV inactivated virus and performed qPCR at 5 and 10 days post infection. We evaluated infected cells for the presence of transcripts detected during latency as well as IE2 mRNA. As expected all transcripts were detected at 1 hr post infection of CD14 (+) monocytes, suggesting that these transcripts are packaged within the HCMV virion (Fig. 5, 1 hr UV). At 5 and 10 days post infection the amount of fold increase for all transcripts from cells treated with non-UV inactivated virus was consistent with what was observed in the RNA-Seq, strongly suggesting lytic infection (Fig. 5, Day 5 and Day 10 UV). However in UV inactivated samples, at 5 or 10 days post infection, all transcripts were below 1-fold increase compared to mock infected samples suggesting that RNA transcripts observed during latency are not the result of input virus or from initial transcription observed at 5 days post infection (Fig. 5, Day 5 and 10 UV). These results indicated that there is a detectable amount of input mRNAs from virions in infected CD14 (+) monocytes, however these input transcripts are short lived and absent by 5-days post infection.

### HCMV UL84 and UL44 interact with the latent HCMV viral genome

The RNA-Seq data identified the presence of two transcripts in HCMV latently infected cells that encode UL84 and UL44, two proteins that participate in virus lytic DNA replication in human fibroblasts [3], [4]. UL84 and UL44 interact with oriLyt and other regions of the HCMV genome during lytic infection [5], [6], [75], [76]. Also, UL84 interacts with UL44, the DNA polymerase processivity factor in infected cells [77]. Since UL44 and UL84 were the only two transcripts encoding apparent DNA binding proteins observed in the RNA-Seq analysis in latently infected cells, it allows for the possibility that these two proteins may participate in HCMV latent DNA replication. The presence of these two transcripts in latently infected cells suggests that maintenance of the latent viral genome may involve some of the lytic virus machinery. Hence we investigated if these two proteins interacted with the viral genome under latent conditions in CD14 (+) monocytes.

ChIP-Seq analysis for UL84 and UL44 was performed in latently infected CD14 (+) cells to determine if these proteins interact with the latent HCMV genome in CD14 (+) cells. Ten million CD14 (+) monocytes were infected with wt FIX BAC virus and mRNA levels were monitored until no IE gene expression was detected (18 days). Infected cells were then processed for ChIP-Seq and DNA-protein complexes were immunoprecipitated using antibodies specific for UL84 or UL44. Immunoprecipitated DNA was used to generate libraries for next generation sequencing using input DNA as a reference. ChIP-Seq was performed using Illumina MiSeq instrument. Data was analyzed and peaks were identified using CLC Bio Genomics Workbench software using the ChIP-Seq analysis package compared to input DNA.

**Figure 6:**
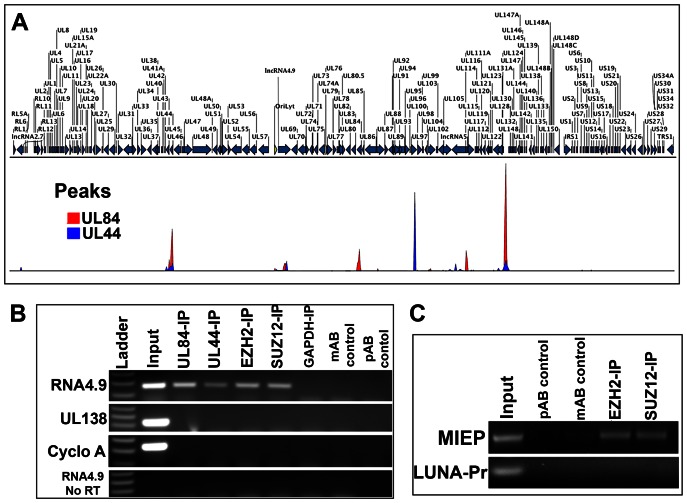
UL44 and UL84 interact with the HCMV latent viral genome in CD14 (+) monocytes. (A) ChIP-Seq analysis peak map of UL44 and UL84 in HCMV latently infected CD14 (+) monocytes. UL44 or UL84 specific antibodies were used to immunoprecipitate protein-DNA complexes followed by next generation sequencing. Shown is the HCMV (Fix strain) virus genome and the location of DNA sequence reads, Red peaks = UL84 protein binding, Blue peaks = UL44 protein binding. (B) RNA4.9 interacts with components for the polycomb repressive complex. HCMV latently infected monocytes were fixed and RNA-protein complexes were immunoprecipitated with antibodies specific for EZH2, SUZ12 or UL84. RNA reverse-transcribed and cDNA was detected by PCR using primers specific for RNA4.9 or Cyclophilin A. Control immunoprecipitations were performed using an isotype control antibodies, mAb control (isotype control for SUZ12 and UL84), pAb control (isotype control for EZH2 and UL44). (C) SUZ12 and EZH2 interact with the MIEP region during latent infection. ChIP assays were performed from latently infected CD14 (+) cells using antibodies specific for SUZ12 or EZH2. Primers specific for the promoter of the MIE gene locus were used as well as control primers specific for the LUNA promoter.

ChIP-Seq analysis identified 5 major peaks corresponding to the binding domains for UL84 in the FIX BAC virus genome during latent infection (Fig. 6A, Red peaks). Two of the peaks mapped to the promoter regions for UL84 and UL44 and suggests that UL84 may regulate the expression of these genes during latent infection (Fig. 6A, Red Peaks.). Another peak was localized to the promoter region for UL112/113 and the upstream regions regulating LUNA and MIE gene expression. Also, the region containing oriLyt and encoding the lncRNA4.9 showed specific binding to UL84 in latently infected cells (Fig. 6A, Red peaks). For UL44, many of the binding domains in latently infected monocytes overlapped with those identified for UL84. UL44 interacted most prominently with the upstream region encoding UL95 (Fig. 6A, Blue peaks), one of the transcripts identified from the RNA-Seq analysis from latently infected CD14 (+) monocytes. Interestingly, UL44 also interacted with the region of the genome encoding lncRNA2.7 and, along with UL84, the region upstream of its own coding sequence (Fig. 6A, Blue peaks). Table 5 is shows the number of reads for UL44 and UL84 ChIP-Seq experiments corresponding to the peak map.

**Table 5:** ChIP-Seq Reads for UL84 and UL44.

UL84 ChIP-Seq		
Gene Promoter	Number of Reads	FDR (%)
UL122/123	1070	6.84×10^−64^
UL44	890	2.00×10^−39^
UL84	217	2.08×10^−07^
UL105/RNA5.0	174	4.13×10^−12^
UL81/82 LUNA	143	0.0012
RNA4.9/UL69	123	5.44×10^−11^
**UL44 ChIP-Seq**		
RNA2.7	366	1.55×10^−44^
UL122/123	311	4.08×10^−10^
UL95	232	0.0797
UL44	214	2.95×10^−6^
UL105/RNA5.0	172	7.66×10^−9^
RNA4.9/UL69	111	0.023
UL112	79	0.098
RNA2.7	25	0.468

These data indicate that UL84 and UL44 interact with the HCMV genome during latent infection in CD14 (+) monocytes and suggests these proteins may activate or suppress several genes during latency and allows for the possibility that viral encoded replication proteins may participate in viral genome maintenance.

### HCMV lncRNA4.9 interacts with UL84 and components of the polycomb repression complex (PRC) during latent infection in CD14 (+) monocytes

Two HCMV transcripts identified in latently infected CD14 (+) monocytes and CD34 (+) cells were long noncoding RNAs. Our previous studies investigating Kaposi's sarcoma-associated herpesvirus (KSHV) lncRNA PAN, showed that herpesvirus encoded lncRNAs can be regulators of both viral and cellular gene expression [62]. lncRNAs associate with chromatin modifying complexes [78] and interact with components of the polycomb repressive complex 2 (PRC2). The PRC2 complex is composed of EZH2, SUZ12 and EED-1. EZH2 is a protein that adds three methyl groups to lysine 27 of histone 3 [79]. SUZ12 is a protein that contains a zinc finger domain that is the point of contact with RNA [80]. EED-1 interacts with HDAC1 and histone deacetylase and various other proteins to mediate gene repression [81]. These PRC2 proteins can mediate changes in histone modifications (methylation) and subsequent repression of gene expression from various genetic loci. Hence the interaction of lncRNAs with PRC2 can globally influence gene expression. Since the RNA-Seq identified HCMV encoded lncRNAs 2.7 and 4.9 during latent infection, we investigated if the newly discovered RNA4.9 could interact with components of the PRC2. Also, since it was previously described that HCMV UL84 is an RNA binding protein [82] and UL84 is also present during latent infection, we also evaluated if lncRNA4.9 interacted with UL84 in latently infected CD14 (+) monocytes.

HCMV latently infected CD14 (+) monocytes were fixed and RNA crosslinking immunoprecipitation (rCLIP) was performed using antibodies specific for EZH2, SUZ12 and UL84. Also, as controls we used an isotype control antibody for immunoprecipitations. Immunoprecipitated RNA-proteins complexes were reverse cross-linked and cDNA was generated. cDNA was used for PCR amplification using primers specific for RNA4.9, cyclophilin A or UL138 RNA. PCR amplification products were observed for immunoprecipitations using UL84, EZH2 and SUZ12 antibodies and PCR primers specific for RNA4.9 (Fig. 6B). No PCR amplification product was detected when the isotype control antibodies or an antibody to the cellular protein GAPDH was used or when using PCR primers specific for UL138 or cyclophilin A (Fig. 6B). Also, no PCR product was observed when the reverse transcriptase was omitted from the PCR protocol (Fig. 6B, RNA4.9, no RT). These results show that HCMV RNA4.9 interacts with viral encoded UL84 and components of the PRC2. Hence, RNA4.9 has the potential to function as a regulatory RNA in the context of HCMV latent infection of CD14 (+) monocytes.

### EZH2 and SUZ12 interact with the MIEP region during latency

**Figure 7:**
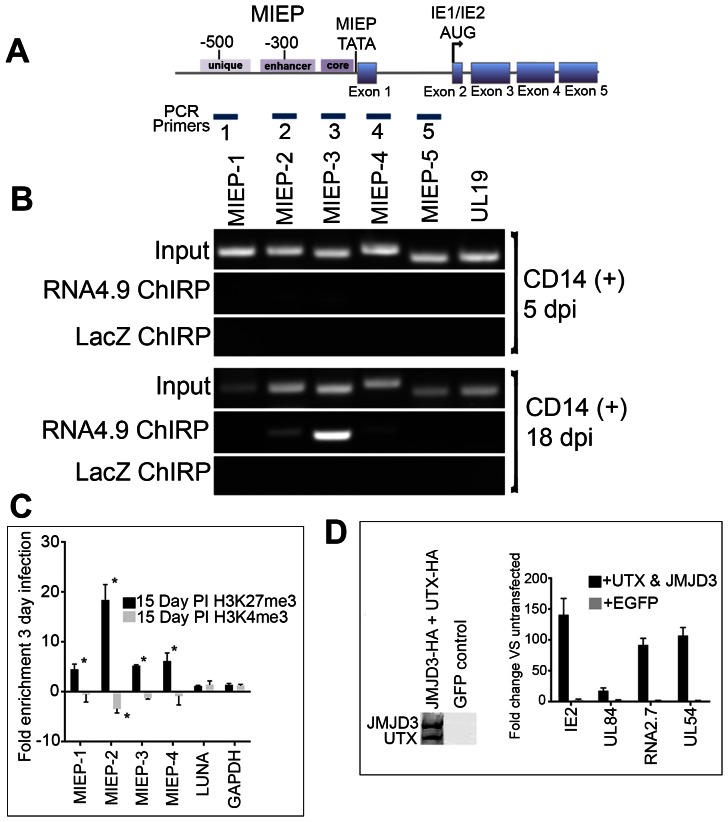
RNA4.9 physically interacts with the HCMV latent viral chromosome. (A) Schematic of the HCMV MIE gene and promoter/enhancer region. Regions amplified by PCR primers are shown that are specific for the unique, enhancer, core promoter, exon 1 and the first intron of IE2. (B) ChIRP analysis of RNA4.9 binding to regions of the MIE promoter and gene locus. Infected CD14 (+) cells were harvested at 5 and 18 days post infection and ChIRP was performed. Control ChIRP was performed using biotinylated hybridization primers specific for LacZ. Amplification of the UL19 region was used as a control for ChIRP PCR amplification. (C) Increase in the enrichment of the repressive H3K27me3 mark at the MIEP during latency is consistent with the binding of RNA4.9. CD14 (+) cells infected with HCMV were harvested at 3-days post infection or during latency at 15 days post infection and ChIP assays were performed using antibodies specific for H3K27me3, H3K4me3 or IgG control antibody. Fold enrichment of H3K27me3 or H3K4me3 was calculated by IgG subtracted % input of each locus divided by the IgG subtracted % input of the control gene GAPDH. Each sample was evaluated in triplicate and the error bars are the SD of the mean. Data is shown as fold enrichment compared to tri methylation state at 3 days PI. Statistical analysis was done using multiple t-test, *P Value<0.001. (D) Increase in IE mRNA accumulation in cells transfected UTX and JMJD3 expression plasmids. Latently infected CD14 (+) cells were transfected with plasmids expressing UTX and JMJD3. Expression of UTX and JMJD3 was confirmed by Western blot, left panel. Graph shows the increase in fold accumulation of specific mRNAs. Error bars are the standard deviation of the average of three experiments.

Since it was demonstrated that RNA4.9 interacts with components of the PRC2 we wanted to evaluate if these same factors bound to the MIEP under latent conditions. We performed ChIP assays using latently infected CD14 (+) cells and immunoprecipitated protein-DNA complexes with antibodies specific for EZH2 or SUZ12. Immunoprecipitated DNA was subjected to PCR using primers specific for the core promoter region for the major immediate early gene locus (PCR primer set MIEP-3, see figure 7 and Table S4 in Text S1). Both SUZ12 and EZH2 were shown to interact with the MIEP region under latent conditions (Fig. 6C). We also evaluated the LUNA promoter during latency for the presence of SUZ12 and EZH2. Consistent with the constitutive expression of LUNA during latency no specific interaction of PRC2 components was observed (Fig. 6C, LUNA-Pr). Control immunoprecipitations using isotype specific antibodies shown no PCR product. These experiments, coupled with the observation that RNA4.9 interacts with polycomb proteins, suggest that RNA4.9 could mediate gene suppression at the MIEP during latency.

### RNA4.9 physically interacts with the latent HCMV genome

As mentioned previously, one of activities of lncRNAs is to mediate changes in gene expression by an interaction with PRC and chromatin. Since we observed the presence of RNA4.9 during latent infection and the transcript was shown to interact with UL84 and PRC2 factors, it is logical to assume that RNA4.9 is involved in regulation of gene expression during latency. The ChIP-Seq analysis showed that the major interaction domain for UL84 was at the MIE promoter (MIEP) region. Therefore we investigated if RNA4.9 interacted with the latent HCMV genome at the MIEP. We performed chromatin isolation by RNA purification (ChIRP) using biotinylated oligonucleotides specific for RNA4.9 to determine if the transcript interacts with the MIEP region during both latent and initial infection of CD14 (+) monocytes. We evaluated the ability of RNA4.9 to interact with the MIEP across 5 regions that mapped to the unique, enhancer, core promoter, exon 1 and the first intron of the IE gene (Fig. 7A). Five and 18 day infected CD14 (+) monocytes were used for ChIRP analysis of RNA4.9 binding to the MIEP. At 5 days, the ChIRP analysis showed only a slight interaction of RNA4.9 with the MIEP and enhancer region (Fig. 7B CD14 (+) 5 dpi, lanes RNA4.9 ChIRP). However during latent infection a strong interaction with the HCMV MIEP, enhancer and a lesser interaction with the first exon was detected (Fig. 7B CD14 (+) 18 dpi, lanes RNA4.9 ChIRP). This interaction, coupled with the observation that RNA4.9 interacts with components of the PRC2 strongly suggest that one mechanism for repression of immediate early gene expression during latent infection is the epigenetic modification of chromatin regulating MIEP region mediated by the virus encoded lncRNA4.9.

Since the interaction of PRC2 with chromatin is associated with the increase in the H3K27me3 repressive mark, we investigated the relative marking of this histone modification at the MIEP region. CD14 (+) cells were infected with FIX BAC virus and cells where harvested at 3-days post infection or during latency at 15 days post infection. ChIP assays were performed using antibodies specific for H3K27me3 or control proteins. The amount of H3K27me3 mark was evaluated for enrichment at MIEP-1, MIEP-2 and MIEP-3 regions (Fig. 7A) as well as at the LUNA and GAPDH promoters. During latency, there was a significant increase in enrichment of the repressive H3K27me3 mark at regions MIEP-2 and MIEP-3 of the MIEP region (Fig. 7C). These loci correspond to the regions where RNA4.9 binding is the most robust (Fig. 7B). The enrichment of H3K27me3 at the LUNA promoter during latency changes only slightly during latent infection, which is consistent with the constitutive expression of LUNA during lytic and latent phases of infection (Fig. 7C). We also evaluated the enrichment of the transcriptional activation H3K4me3 mark at the MIEP region. This mark does decrease during latent infection, consistent with less gene activation (Fig. 7C). The results of these experiments suggest that RNA4.9 interacts with the MIEP region and mediates the enrichment of the repressive H3K27me3 mark to repress HCMV transcription of IE2.

Since we observed the enrichment of the repressive H3K27me3 mark at the MIEP region we investigated if the over expression of specific H3K27me3 demethylases could have an affect on reactivation, specifically the release of repression of IE2 gene expression. Plasmids expressing UTX and JMJD3 were cotransfected into latently infected CD14 (+) monocytes and 72 h post transfection total cellular RNA was harvested and qPCR was performed. We examined mRNA accumulation for IE2, UL84, RNA2.7 and UL54 (polymerase). As a control, we transfected a plasmid that expressed EGFP. mRNA expression levels were compared to mock transfected latently infected CD14 (+) cells. In cells cotransfected with UTX and JMJD3 expression plasmids there was a 130 fold increase in IE2 mRNA accumulation (Fig. 7D). Transcripts encoding RNA2.7, UL84 and UL54 also increased markedly in cells transfected with demethylases expressing plasmids (Fig. 7D). Latently infected CD14 (+) cells transfected with the EGFP expressing plasmid show no significant increase in mRNA accumulation (Fig. 7D). These experiments strongly suggest that latent HCMV genomes are silenced, at least partly, by repressed chromatin marked with H3K27me3. Also, our data suggests that RNA4.9 participates in transcriptional suppression of MIE gene expression.

### Identification of nucleosome depleted regions within the HCMV genome during latent infection of CD14 (+) monocytes

**Figure 8:**
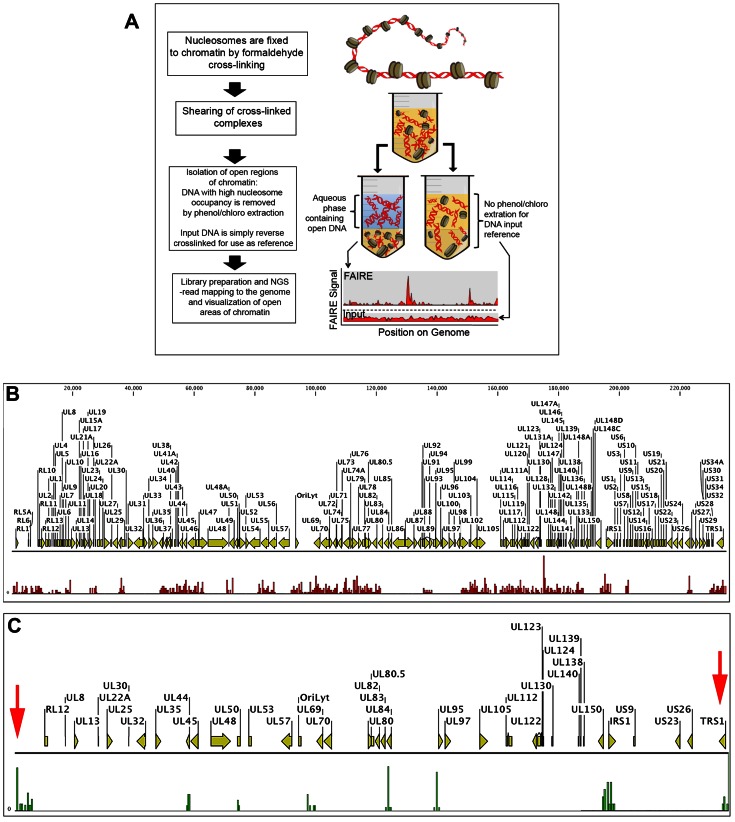
Analysis of nucleosome depletion in HCMV infected CD14 (+) monocytes. (A) Schematic of FAIRE method. FAIRE-Seq was performed using 4 or 18 day infected CD14 (+) monocytes. (B) FAIRE-Seq analysis of 4 day post infection of CD14 (+) monocytes. (C) FAIRE-Seq analysis of 18 day latently infected CD14 (+) monocytes. Sequencing reads were mapped to VR1814. Red Arrows indicate nucleosome depletion at the TR region of the HCMV genome.

We postulate that the region(s) of the viral genome that could potentially serve as latent replication/maintenance elements should be depleted of nucleosomes and hence would define the major cis regulatory elements within the latent HCMV chromosome. This assumption is based on previous studies showing that origins of replication are depleted of bulk nucleosomes [68], [83]–[86]. It was further demonstrated in *Saccharomyces cerevisiae* that nucleosomes are positioned as flanking replication origins [87]. We employed the method **F**ormaldehyde **A**ssisted **I**solation of **R**egulatory **E**lements (FAIRE) to determine which regions within the latent HCMV chromosome are depleted of nucleosome structures [88]–[90]. FAIRE is a powerful approach to identify genome-wide active regulatory elements *in vivo*. FAIRE involves formaldehyde crosslinking of cells, followed by shearing of chromatin and subsequent phenol/chloroform extraction. The procedure is based on the fact that histones will crosslink with DNA and nucleosome free chromatin will be preferentially partitioned into the aqueous phase. These genomic regions are then mapped back to the HCMV genome using next generation sequencing (Fig. 8A).

To study the HCMV chromosome during latent infection we used the CD14 (+) monocyte experimental model [46]. In this experimental latency model, CD14 (+) monocytes are cultured using specific growth media and culture plates that retain CD14 (+) monocytes in an undifferentiated state. Cells were infected with the HCMV clinical isolate FIX BAC strain and monitored for the expression of immediate early gene expression and the presence of the previously described latency associated transcripts UL138 and LUNA [41], [53], [91]. At 14 days post infection of CD14 (+) monocytes, the level of IE1/2 mRNA was undetectable by qPCR, however UL138 and LUNA transcripts were still present (not shown).

Latently infected cells were harvested and subjected FAIRE followed by next generation sequencing. CD14 (+) monocytes were infected with FIX BAC virus and cells were harvested at 4 and 18 days post infection. At 18 days post infection cells expressed latency-associated transcripts and were absent for expression of immediate early transcripts. Cells were subjected to FAIRE and DNA was analyzed using next generation sequencing (60 million reads per sample, paired end sequencing). FAIRE data was analyzed using CLC Bio Genomics Workbench software (ChIP-Seq analysis). At 4 days post infection of CD14 (+) monocytes over 130 peaks or nucleosome-depleted regions were elucidated (Fig. 8B). These regions were distributed across the HCMV genome with a prominent peak at the major immediate early promoter region (Fig. 8B). These “open” active regions across the HCMV genome are consistent with robust transcription and replication during lytic infection. The FAIRE profile was significantly different during a latent HCMV infection at 18 days. Nucleosome depleted regions of the viral genome were consistent with what was observed from the RNA-Seq analysis (Fig. 2B), in that active regions were specific for loci of the viral chromosome where latent viral transcription was observed (Fig. 8C). One obvious exception was the high degree of sequence reads detected for the inverted and terminal repeat region (TR) of the genome (Fig. 8C, Red Arrow). Although nucleosome depleted regions were identified at both IR and TR regions of the genome, next generation sequencing could not distinguish between the two regions because of the high degree of homology. Also, although a portion of the IR region is not present in FIX BAC, the IR region was identified because the FAIRE-Seq reads were mapped to the annotated parent virus strain (VR1814), hence the nucleosome depleted regions were localized only to the TR regions of the genome. The presence of these highly nucleosome depleted loci, coupled with the observation that no viral transcripts were detected from this region by RNA-Seq during latent infection, strongly suggests that these regions of the genome could act as elements that mediate DNA replication/maintenance.

### HCMV terminal repeat region interacts with cellular encoded DNA replication licensing factors

**Figure 9:**
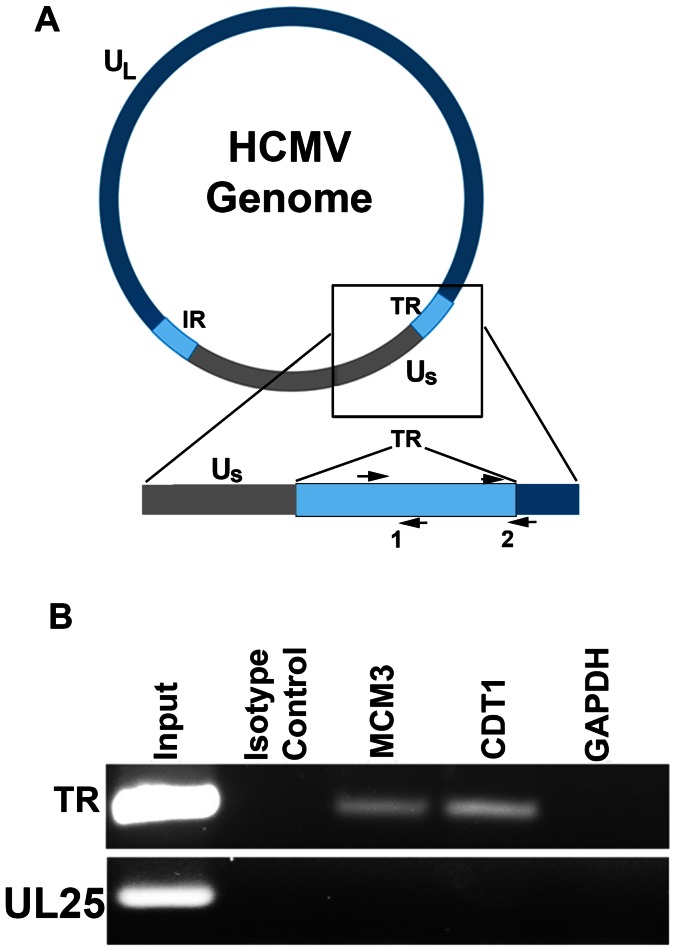
Interaction of MCM3 and CDT1 with HCMV TR DNA sequences in latently infected CD14 (+) monocytes. Latently infected cells were treated with formaldehyde and subjected to ChIP using antibodies specific for MCM3 or CDT1. (A) Schematic of the HCMV genome showing the terminal repeat (TR) region. Also shown are primer sets specific for the TR segment of the genome (1 and 2). (B) ChIP assay showing an interaction of the TR region of the genome with MCM3 and CDT1 in latently infected cells. Control immunoprecipitations were the use of an isotype antibody control and an antibody specific for GAPDH. Control PCR primers were specific for the HCMV UL25 ORF.

Since FAIRE data strongly suggested that the TR region was depleted of nucleosomes we investigated if the TR region associated with the protein components of the pre-replication complex (pre-RC). HCMV latently infected CD14 (+) monocytes were fixed and a chromatin immunoprecipitation (ChIP) assay was performed using antibodies specific for pre-RC proteins MCM3 and CDT1 proteins. We also performed immunoprecipitations using isotype control antibodies and an antibody to GAPDH. Figure 9A is a schematic showing the HCMV genome and the location of the terminal repeat (TR) region of the viral DNA. Also shown are primer sets used for amplification of DNA immunoprecipitated from the ChIP assay. Immunoprecipitated protein-DNA complexes were amplified using PCR primers designed to amplify the TR (Fig. 9A, set 1 and 2) of the HCMV genome. As a control we also used primers specific for the UL25 genomic locus. PCR products were observed for ChIP samples using CDT1 or MCM3 specific antibodies when primer set 1 was used in the PCR mixture (Fig. 9B). No specific amplification product was observed from samples using isotype control or GAPDH antibodies, or using primers designed to amplify the UL25 region (Fig. 9B). These data strongly suggest that the TR region of the HCMV genome interacts with factors involved in cellular DNA replication and is consistent with previous results from other herpesvirus systems and latent origins.

### A plasmid clone containing the HCMV TR element persists in latently infected CD14 (+) monocytes

**Figure 10:**
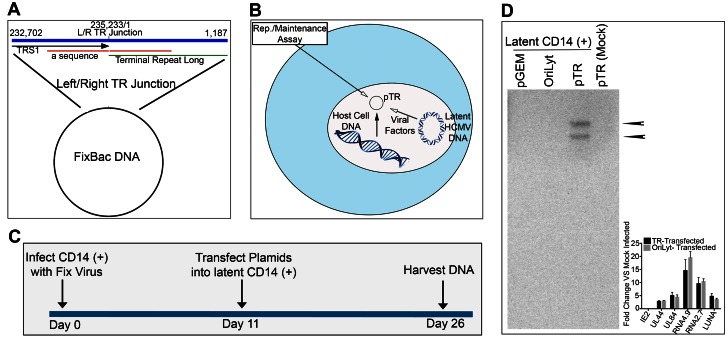
A plasmid clone containing the circularized TR region of HCMV genome is maintained in latently infected CD14 (+) monocytes. (A) Schematic showing the circularized region of the HCMV genome subcloned into the plasmid vector pGEM7zf(−). (B) Schematic showing the development of a latent replication/maintenance assay in CD14 (+) monocytes. (C) Infection/transfection protocol used to evaluate plasmid maintenance in latently infected CD14 (+) monocytes. (D) Southern blot of a Gardella gel containing samples from latently infected CD14 (+) monocytes transfected with plasmids pGEM7zf(−), pOriLyt, pTR or pTR from uninfected CD14 (+) cells 26 days post transfection. Arrows indicate the presence of two bands in the pTR lane at 15 days post transfection, 25 days post infection.

Since our data suggested that the terminal repeat region of the HCMV genome was involved in replication/maintenance of the latent viral genome, our next step was to develop an assay to evaluate the ability of the TR element to mediate genome maintenance in latently infected cells. We postulate that if the TR element mediates viral chromosome maintenance then a plasmid containing the TR element would persist in latently infected cells. To this end we subcloned the HCMV FIX BAC TR element, as it would exist in the circular genome form (Fig. 10A), since previous data indicated that latent HCMV genomes exist as a circular episome [92]. This TR subclone, pTR, would be used to transfect HCMV latently infected CD14 (+) monocytes where required viral and cellular encoded factors would be supplied in *trans* from resident viral DNA and the host chromosome (Fig. 10B). Latently infected CD14 (+) monocytes were transfected with pTR using Amaxa nucleofector and cells were processed as shown in figure 7C. As controls, we also transfected the parent vector pGEM and a plasmid that contains the HCMV origin of lytic DNA replication, oriLyt. After 15 days post transfection (26 days post infection) cells were prepared and DNA was resolved using a Gardella gel [63]. The gel was transferred to a nylon membrane and hybridized to a radiolabeled pGEM probe. Several bands were detected in the lane that contained cells that were transfected with the TR-containing plasmid whereas lanes that contained samples that were transfected with either pGEM, cloned oriLyt or mock infected TR transfected cells failed to show a hybridization product (Fig. 10D). Two bands were detected on the Southern blot from cells containing pTR. These two bands could be due to variable lengths of the repeat sequence present with the TR region of the genome. We also evaluated 26 day latently infected CD14 (+) that were transfected with oriLyt or TR containing plasmids. All transfected cells expressed latency-associated transcripts, in the absence of IE2 gene expression, detected in the RNA-Seq analysis at 26 days post infection (Fig. 10D, inset graph).

These data show that the cloned HCMV TR element is capable of persisting in latently infected CD14 (+) monocytes. This is the first report describing the existence of a DNA element within the HCMV genome that mediates maintenance of the viral chromosome during latency.

## Discussion

To date HCMV latency is defined as the lack of production of infectious virus, absence of immediate early gene expression and the presence of a few specific latency-associated transcripts, as well as the ability to reactivate latent resident viral DNA. Previous studies have identified the expression of some latency-associated transcripts in both naturally and experimentally infected CD14 (+) and CD34 (+) cells [41], [42], [48], [49].

We utilized two experimental systems to study HCMV latency; the first system developed for CD14 (+) monocytes is where cells are cultured in an undifferentiated state by using specific cytokines and a growth surface that retards cell attachment [46]. Using CD14 (+) monocytes cultured under these specific conditions resulted in the presence of the HCMV genome in the absence of immediate early gene expression after approximately 18 days post infection. For the HCMV latency infection protocol used here, we treated CD14 (+) monocytes with media formulation previously shown to maintain monocytes in an undifferentiated state [46]. Although it was previously reported that IE gene expression was not detected at 11 days post infection, in our hands the loss of IE gene expression required the culturing of cells for approximately 14 days. Hence, to ensure that viral infection was indeed in a latent phase, we evaluated transcription at 18 days post infection and only when cells were negative for IE gene expression. RNA-Seq has major advantages over other previously employed methods to identify HCMV latency associated transcripts. RNA-Seq allows for a quantitative unbiased evaluation of transcripts present in infected cells. After a five-day infection, RNA-Seq showed the presence of transcripts originating from most of the HCMV genome. Although this observation suggests that a lytic infection precedes the establishment of latency, more experiments are needed to confirm this finding. Previous studies demonstrated that viral gene expression was required for establishment of latency and several virus-encoded genes were identified [39], [48]. Although at present we do not know the significance of wide spread gene expression in CD14 (+) at early time points post infection, a lytic-type infection upon initial infection of primary cells was observed previously for KSHV and EBV [93]–[95]. In those systems, this initial burst of lytic replication may be critical for subsequent establishment of latency. The observed lytic infection for HCMV in monocytes warrants further investigation and will be the focus of future studies.

The second experimental latency system used infection of CD34 (+) cells where we isolated CD34 (+) cell populations, since evidence suggests that these cell types support HCMV latent virus [96]. RNA-Seq showed that infected CD34 (+) cells did not undergo the same robust HCMV viral gene expression pattern as observed with initial infection of CD14 (+) monocytes. At three days post infection of CD34 (+) cells only a subset of genes were expressed, whereas in CD14 (+) cells almost the entire HCMV genome showed active transcription. Although the same core transcripts, including the lncRNAs RNA2.7 and RNA4.9, were detected in latently infected CD34 (+) cells and CD14 (+) monocytes, some differences were also observed. One of the most notable was the presence of IE1 mRNA in latently infected CD34 (+) cells, which was also confirmed in naturally infected CD34 (+) cells. The presence of IE1 mRNA in latently infected CD34 (+) cells was observed previously [26] and allows for the possibility that this protein could tether the HCMV chromosome to genomic DNA since it was previously demonstrated that IE1 interacts with cellular DNA [97], [98]. The lack of detection of IE2, late gene expression or early genes involved in lytic DNA replication argue against the possibility that that a population of cells are undergoing lytic replication.

Previous studies have examined the expression patterns in HCMV latently infected cells. All of these previous studies used microarrays to evaluate the presence of mRNAs [26], [41], [48]. Although these studies yielded important information regarding gene expression during latent infection, RNA-Seq has several advantages over microarrays [99]–[101]. One major advantage is that RNA-Seq is unbiased in that no prior knowledge about mRNA sequence is required. One example of this demonstrated in the present study is that most microarrays target known ORFs, hence the presence of expressed lncRNAs or antisense transcripts may be overlooked. Also, recent studies indicate that RNA-Seq is more sensitive than conventional microarrays [102], [103].

One of the most striking findings from the study presented here is the observation that transcripts that encode proteins associated with lytic DNA replication were expressed during latency. These transcripts were present even though immediate early mRNAs were not expressed after approximately 14 days post infection of CD14 (+) monocytes. Hence the observed expression of transcripts identified was not the result of transactivation from IE2. One other explanation for the presence of apparent lytic mRNAs is the detection of transcripts with long half-lives that were still present from the initial infection and lytic replication. This is unlikely since infection of CD14 (+) monocytes with UV-inactivated virus indicated that, although all latent RNAs were detected at 1 hr post infection due to apparent packaging in virions, we were unable to detect these transcripts at 5 or 10 days post infection. This suggests that these mRNA half-lives are less than 5 days in CD14 (+) monocytes.

The RNA-Seq analysis showed that mRNAs encoding ORFs UL50 and 52 were present in latently infected cells. The expression of these ORFs during latency is interesting since UL52 is implicated in cleavage-packaging. UL52 a protein required for virus growth in human fibroblasts is localized to the nucleus and appears to enclose replication compartments. Although implicated in encapsidation/cleavage of virus DNA, UL52 was not associated with other proteins known to perform these functions [104]. However, UL52 is quite unique with respect to other proteins involved in cleavage and packaging in that it is localized to the nucleus and found in replication compartments [104]. Hence, it could be that UL52 supplies a function in latency that is related to replication of the virus genome during latent infection.

For UL50, this protein is implicated in nuclear egress and is associated with the nuclear lamina as part to the nuclear egress complex (NEC) [105]. UL50 associates with the cellular factors p32 and protein kinase C (PKC). UL50 is localized to the inner nuclear membrane and associated with the nuclear lamina along with UL53 [105], [106]. UL50 was shown to associate with BiP and this interaction was essential for phosphorylation of the nuclear lamina [106]. UL50 along with UL53 may act to remodel the nuclear lamina [107]. We speculate that UL50, like UL52, may play a role in maintaining the integrity of the HCMV latent virus genome.

In both latently infected CD14 (+) and CD34 (+) cells the transcripts encoding UL95 and UL87 were among the most abundant. This observation also occurred in naturally infected cells. UL87 and UL95 encode essential early transcripts that produce proteins that apparently affect late gene expression including the UL44 late kinetic transcription and colocalize with UL44 prior to initiation of viral DNA synthesis [108]. Interestingly, the pre expression of UL87 inhibited the expression of MIE genes and virus DNA replication [108]. UL87 and UL95 are recruited to replication compartments during lytic infection [108]. Hence, during latent infection UL87 and UL95 could serve to enhance the expression of UL44 and/or UL50 and 52. Another possibility is that UL87 and UL95 may serve to help suppress the expression of IE2 during a latent infection.

UL84 is the putative initiator protein for lytic DNA replication and interacts with UL44 [5], [76], [82], [109], [110]. UL84 is a phosphoprotein that exhibits nucleocytoplasmic shuttling that is required for function [82], [111]. UL44 is the DNA polymerase processivity factor, however these herpesvirus proteins have recently been shown to have diverse functions with respect to regulation of gene expression and initiation of DNA synthesis [112]–[117]. The observation that transcripts encoding UL44 and UL84 are produced in latently infected cells suggests that the maintenance of the HCMV virus genome may involve a mechanism that utilizes some of the HCMV lytic DNA synthesis machinery. UL84 interacts with both Ku70 and Ku80 and the Ku70/80 complex is involved in DNA repair [76]. Ku70/80 is and ATP dependent helicase that is involved with DNA repair and interacts directly with the RNA component (hTR) of telomerase [118], [119]. These interactions suggest that stability of the HCMV genomic DNA in latently infected cells may also involve DNA repair enzymes and telomerase. We also demonstrated that UL84 protein interacted with the HCMV latent genome. We speculate that UL84 may act to suppress IE gene expression, however UL84 may also mediate its own expression and that of UL44 and LUNA. The interaction of UL84 with oriLyt could mean that the protein acts to suppress the activation of the lytic replication. However, it is just as plausible that UL84 (possibly in cooperation with cellular factors) activates the promoter region within oriLyt to facilitate the expression of RNA4.9. Another possibility could be that UL84 is acting in the capacity of a replication factor at oriLyt to replicate the latent genome. Interestingly, UL84 is dispensable for lytic DNA replication in one clinical isolate, BACmid clone (TB40/E) [120]. Hence one possibility is that in clinical isolates UL84 is not required for lytic replication, but may mediate latent genome maintenance.

LncRNAs have emerged as significant regulators of gene expression in human cells. Although the mechanisms used by lncRNAs vary, one well-defined mechanism involves their interaction with chromatin modifying complexes. lncRNAs can act as molecular scaffolds to mediate epigenetic changes in histones, which results in activation or suppression of gene expression [121]–[124]. Hence, lncRNAs can globally affect gene expression patterns. We have recently defined one mechanism of action for KSHV PAN RNA in lytically infected cells. PAN RNA is a highly abundant transcript and we show that expression of PAN RNA can result in the disregulation of genes involved in immune response and cell cycle [62]. This suppression of gene expression is most likely achieved by the interaction of PAN RNA with polycomb proteins. Activation of KSHV and cellular gene expression by PAN RNA appears to involve its interaction with the demethylases UTX and JMJD3, which remove the repressive H3K27me3 mark [62]. Therefore evidence shows that viral lncRNAs are significant regulators of both cellular and viral gene expression.

In HCMV, the lncRNA2.7 was shown to regulate the apoptosis pathway during lytic infection [125]. Since we also observed the expression of RNA2.7 in latently infected cells it is likely that this transcripts plays the same role in latently infected CD14 (+) cells. Early studies did not detect the presence of this transcript in latently infected CD14 (+) cells [35]. One possibility for the discrepancy between this early study and the present one is that we used highly sensitive RNA-Seq, where the early studies used traditional RT-PCR.

The lncRNA4.9 was recently discovered by next generation sequencing of the HCMV transcriptome during lytic infection [10]. RNA4.9 initiates within oriLyt and extends upstream and terminates just downstream of UL69 [10]. Hence, since of the location of the 5 prime start of transcription for RNA4.9 is within oriLyt this suggests that the promoter region is also within oriLyt. We previously identified a bidirectional promoter within oriLyt just upstream of the RNA4.9 transcriptional start site [126]. We now show data that strongly suggests that RNA4.9 may act as a regulatory RNA with the potential to control cellular and viral gene expression during HCMV latency in CD14 (+) monocytes. This is the first reporting of an HCMV encoded RNA that interacts with the PRC. Using ChIRP we show that RNA4.9 physically interacts with the HCMV latent genome in the MIEP region. This observation, combined with the data showing that RNA4.9 interacts with PRC proteins that are also bound to the same locus suggests that this transcript may repress the expression of IE2 during latency. Previous studies have demonstrated epigenetic regulation of IE gene expression [27], [37], [47], [127]. These earlier reports investigated the deposition of the acetylated H4 mark within the MIEP. PRC2 is associated with the repressive H3K27me3 mark; hence we examined the presence of this mark at the MIEP. Recent studies have shown that the presence of H3K27me3 is associated with herpesvirus latent genomes and repression of lytic gene expression [128]–[131]. One of the key factors for mediating gene silencing is lncRNAs [132], [133]. Data presented here strongly implicates lncRNA4.9 as a targeting factor for suppression of IE gene expression during latent infection in CD14 (+) cells.

For CD34 (+) latently infected cells, the presence of UL28/29 and UL37/38 is interesting. It was recently reported that this locus encodes a spliced transcript and stimulate the accumulation of immediate early RNAs [134]. UL28/29 proteins interact with nucleosome remodeling and deacetylase protein complex, NuRD along with UL38 and UL28/29 enhanced activity of the MIEP [135]. Recently, UL28/29 was shown to interact with UL84, p53 and suppresses cellular gene expression during lytic infection [136]. Hence the expression of UL28/29 during latency suggests viral control of p53-regulated genes in the context of a latent infection.

Although RNA-Seq showed some differences between the CD34 (+) and CD14 (+) latent transcriptome, many transcripts were common to both systems including the presence of lncRNAs, UL84 and UL44. One explanation for the presence of different factors could be that specific viral factors may be required to maintain genome latency in a cell type specific manner.

Our RNA-Seq evaluation of HCMV naturally latently infected CD14 (+) and CD34 (+) cells isolated from seropositive donors showed an almost exact match to the transcripts observed from experimental latency, although relative abundances differed. Since pooled blood from HCMV seropositive donors was used, we do not know the prevalence or degree of transcript expression in individual donors. Nevertheless, data shows that experimental latency closely matches natural latent infection. This is the first evaluation of natural latent infection by next generation RNA sequencing. Interestingly, RNA-Seq detected the previously transcript originating from UL126a in naturally infected cells [137], [138]. This transcript was not detected in our experimental latency model. One explanation for the discrepancy is that some differences between natural and experimental latency may exist. This could be due the fact that natural infection data was obtained from pooled donors and hence variations in expression levels are reflected in the RNA-Seq analysis. Nevertheless, overall the transcripts present during natural and experimental infection vary little. This is a very significant finding in that HCMV CD14 (+) and CD34 (+) experimental latency systems are widely used. Our data suggests a strong link between transcripts expressed during HCMV natural latent infection and those that occur in cell culture.

It was previously demonstrated that herpesvirus latent origins are marked by lack of nucleosome structure and the presence of cellular factors involved in DNA replication or licensing [139]. Also, it was shown that nucleosome assembly proteins interact with herpesvirus-encoded proteins to regulate replication at the latent origins replication [140], [141]. For EBV, EBNA-1 was shown to destabilize nucleosomes at the latent origin [142]. Also, for EBV, cellular factors play a significant role in replication of the latent virus origin [143]–[145]. Hence previous evidence indicates that specific herpesvirus and cellular proteins bind to DNA domains to mediate latent viral genome replication and maintenance. Currently little data exists regarding the mechanism involved in maintenance of the viral chromosome in HCMV latently infected cells. Previous studies indicated the HCMV latent genome is in a circular form [25]. Based on the latency models from the gamma herpesviruses, it is assumed that the maintenance and replication of the HCMV latent viral genome requires trans and cis acting factors. In this report we show that, consistent with the presence of active chromatin, that the TR region of the HCMV genome are depleted of nucleosomes during latent infection of CD14 (+) monocytes. We developed a latent replication/maintenance assay where CD14 (+) monocytes harboring the latent HCMV chromosome were transfected with the TR containing plasmid. Control plasmids, including a plasmid that contained oriLyt, failed to persist in latently infected cells. The TR element of HCMV in the circular form contains two “a” sequences and many repetitive DNA sequence motifs. Hence, during replication/maintenance there is a potential for variability of these repeat sequences. The fact that we observed two bands in the Gardella gel may indicate the presence of variable repeated regions. This was also postulated for the KSHV latent origin, which also contains several repeated DNA sequences [15]. Although the Gardella gel clearly shows that the TR plasmid can persist in latently infected cells, further studies are needed to demonstrate if the TR element can direct plasmid replication. Nevertheless, this is the first reporting of a region of the HCMV chromosome that mediates viral DNA maintenance.

The identification of cis and trans acting factors involved in HCMV latency in CD14 (+) monocytes and CD34 (+) cells now allows for a more in depth analysis of the factors required for viral chromosome maintenance/replication.

## Supporting Information

Text S1 Supporting tables: S1. qPCR primers and probes. S2. ChIRP primers. S3. Primers used for RT-PCR. S4. Primers used for H3K27me3 evaluation.
(DOCX)
