# Supplementary material for: Cis and Trans Acting Factors Involved in Human Cytomegalovirus Experimental and Natural Latent Infection of CD14 (+) Monocytes and CD34 (+) Cells
Source: PLoS Pathog. 2013 May 23;9(5):e1003366. doi: 10.1371/journal.ppat.1003366 (PMC3662700; doi:10.1371/journal.ppat.1003366)
Supplement: Text S1 — Supporting tables: S1. qPCR primers and probes. S2. ChIRP primers. S3. Primers used for RT-PCR. S4. Primers used for H3K27me3 evaluation. (DOCX) [file ppat.1003366.s001.docx]

| **Supplemental Table S1. qPCR primers and probes.** |
| --- |
| **IE1/IE2** |
| Forward: CCAAGAGAAAGATGGACCCTG |
| Reverse: AACATAGTCTGCAGGAACGTC |
| Probe: /56-FAM/CCCGAGACA/ZEN/CCCGTGACCAAG |
| **IE2** |
| Forward: AAG ATG CGC ACC ATG ACC TGT TTG |
| Reverse: AGC CTC AAA GAA TTG CAC ACC CAC |
| Probe: /56-FAM/TTA CCG CAA /ZEN/CAT GAT CAT CCA CGC TG/3IABkFQ/ |
| **UL84** |
| Forward: TTT CTT CTT CTT GCA ACG TCG CGG |
| Reverse: AGA ATT GCG GGA TCC CTT CCA GAT |
| Probe: /56-FAM/TCT GCT CTC /ZEN/TAC GCC GCT GCA ATT /3IABkFQ/ |
| **UL44** |
| Forward: CAG CTG CAC GTT GAT ACG CAT GTT |
| Reverse: TCC ACC GGC CAT CAA GTT TAT CCT |
| Probe: /56-FAM/ACA GCC AAT /ZEN/AAC CGC GTC AGT TTC CA/3IABkFQ/ |
| **UL81-82ast (LUNA)** |
| Forward: ATC AGG CCG TTC ATT TGG AAC ACC |
| Reverse: ATG ACG TTG CTC CGT GGA AAG AGA |
| Probe: /56-FAM/TTT GTC CGG /ZEN/TTT ACC GGG TGT GAG A/3IABkFQ/ |
| **RNA4.9** |
| Forward: CTG TTC CGT GAT GCT ACC TAG |
| Reverse: AGC ACG AGA TGG TTT GAC G |
| Probe: /56-FAM/CG ACT CTT C/Zen/C TGT TTT CGC CCT GA/3IABkFQ/ |
| **UL105** |
| Forward: GCA CAT GTA CAC TAC CTT CCG |
| Reverse: TGA TCT GAC AGT TGG CCT TG |
| Probe: /56-FAM/ACG TTG CCG/ZEN/CTCGTTACCTACAATC/3IABkFQ/ |
| **UL138** |
| Forward: TCC ACG TCG CTA ACC AGA GAA ACA |
| Reverse: AAT GTA CCA TGG CTA CGG TGG TGA |
| Probe: /56-FAM/ATC TGT TGA/ZEN/AAC CCG TGA CGG GAT/3IABkFQ/ |
| **UL54** |
| Forward: GTG CGC GAT CTG TTC AAC ACC ATT |
| Reverse: TGC TGT AGT GGT TGG GCA GGA TAA |
| Probe: /56-FAM/ATT CCG TTG /ZEN/CGG CGT GTC ATC TTT GA/3IABkFQ/ |
| **UL95** |
| Forward: ATT TGC CTT TAA AGC CCG TCT CGC |
| Reverse: TCA CGC GCG AGA TCT AAA CAG AGA |
| Probe: /56-FAM/TGT TTG TTG /ZEN/TGC GCG CTC TAC AGT CA/3IABkFQ/ |

| \| **Oligo name** \| \| --- \|  \| #1-RNA 4.9 \| \| --- \| \| #2-RNA 4.9 \| \| #3-RNA 4.9 \| \| #4-RNA 4.9 \| \| #5-RNA 4.9 \| \| #6-RNA 4.9 \| \| #7-RNA 4.9 \| \| #8-RNA 4.9 \| \| #9-RNA 4.9 \| \| #10-RNA 4.9 \| \| #11-RNA 4.9 \| \| #12-RNA 4.9 \| \| #13-RNA 4.9 \| \| #14-RNA 4.9 \| \| #15-RNA 4.9 \| \| #16-RNA 4.9 \| \| #17-RNA 4.9 \| \| #18-RNA 4.9 \| \| #19-RNA 4.9 \| \| #20-RNA 4.9 \| \| #21-RNA 4.9 \| \| #22-RNA 4.9 \| \| #23-RNA 4.9 \| \| #24-RNA 4.9 \| \| #25-RNA 4.9 \| | \| **Probe (5'-> 3')** \| \| --- \| \| gcgtactgacgacaattgaa \| \| ttaagggacacggttttgtc \| \| tttgaacagagaaaggtggg \| \| ttcgctctgagcagaaaaaa \| \| aggtgttcttttcttcgagc \| \| ttcgtctacgtggtaagagt \| \| aaacagatgcaaatcgccta \| \| aaaaatataggtgccggacg \| \| atggagggatatcaggtcat \| \| cacatttctagtcgtgacgg \| \| gtcgattcagaccgacaaaa \| \| caaacgatggtaagaggtcg \| \| taccgatagagcctgagatg \| \| tccggaaagatggtgttttt \| \| atagatccagagagcgtacc \| \| gatgatgtggttcgtcgtac \| \| accagagactaagtcggaaa \| \| ctgtgacggtgattcttcag \| \| gtgcatcatacaacgacact \| \| ttcggtagagagctacagtc \| \| gacgggaatcgatgtcattt \| \| ctatgtacaagacgcatggg \| \| ggtggtagtacaagggtttg \| \| gtgacgatgatgatgtggtt \| \| ttttggttcgaatcgagctt \| |
| --- | --- | --- | --- | --- | --- | --- | --- | --- | --- | --- | --- | --- | --- | --- | --- | --- | --- | --- | --- | --- | --- | --- | --- | --- | --- | --- | --- | --- | --- | --- | --- | --- | --- | --- | --- | --- | --- | --- | --- | --- | --- | --- | --- | --- | --- | --- | --- | --- | --- | --- | --- | --- | --- |

**Supplemental Table S2. ChIRP primers**

| **Supplemental Table S3. Primers used for RT-PCR.** | | |
| --- | --- | --- |
| **Gene** | **Forward** | **Reverse** |
| RNA Beta2.7 RT | AGATGAAATTATCCCGTGTCCG | GTGAAATCTGGCTTGGTTGTG |
| UL13 RT | AAATTCAACGCACACTCAACG | CTATTCAGTTCTCCTTGTCCCG |
| UL14 RT | GGATTACTGGCTGACGGATC | TTTCCTCTTCGGTGTTCAGAC |
| UL44 RT | TGCACGTGGATCTAGATTTCG | ACTGACACGGTTATTGGCTG |
| UL50 RT | CGGTCTATTGTGTCGAGTATCTG | AGCACATTGAACTCACCTACG |
| UL52 RT | TGTCCGAGTTTACGCATCTG | TGGTTTAGGGTGACATTCTCG |
| RNA4.9 RT | ATTCCCCACCTTTCTCTGTTC | CTCTCGACGCCATTTTCTCT |
| UL79 RT | AGCGTACGAACCCTTGATTC | GTGAGAAAGAGGAGGATGGG |
| LUNA UL8182ast RT | TCCTCTTCTTCCTCCTCTTCC | TGGGTTTTGGACTCTCACAC |
| UL84 RT | TCAAATCCACGCTACACTCG | CAGGACTCGTTTTCTCTGTCG |
| UL87 RT | CAATTCGTACCAGATCCCCTTC | CGTGAGGTTCTTGCTGTAGTC |
| UL95 RT | TTTCAACGTCCCTGTGCTC | GCTACTGCTATCCAACACCG |
| IE286 RT | GACCCTGACAACCCTGACGAG | TTGCGGTACTGGATGGTAAAG |
| UL138 RT | GATGAGATCTTGGTCCGTTGG | TCACGGGTTTCAACAGATCG |
| US21 RT | AATGATCTCCATCGTGTGCC | GCGTGAGGAAACAAAATAGCG |
| Cyclophilin A RT | CACCGTGTTCTTCGACATTG | ACCCTGACACATAAACCCTG |

**Table S4. Primers used for H3K27me3 evaluation.**

| **GAPDH** |
| --- |
| Forward: CCAAGAGAAAGATGGACCCTG |
| Reverse: TCGAACAGGAGGAGCAGAGAGCG |
| **LUNA** |
| Forward: AATAGCGACGGTGGAAGTG |
| Reverse: GCAAACAGATCGCGTTTCAG |
| **MIEP-1** |
| Forward: CGAAACTGCGATATTTGCGAC |
| Reverse: TTGGGCATACGCGATATCTG |
| **MIEP-2** |
| Forward: GGCCATTTACCGTCATTGAC |
| Reverse: TCAATGGGTGGAGTATTTACGG |
| **MIEP-3** |
| Forward: CGGTACTTACGTCACTCTTGG |
| Reverse: ACGCTGTTTTGACCTCCATAG |
| **MIEP-4** |
| Forward: GGAGATCCCACGCTATGTTT |
| Reverse: AGACTGACACGGACTCTGTAT |
